# Landscape characteristics shape surface soil microbiomes in the Chihuahuan Desert

**DOI:** 10.3389/fmicb.2023.1135800

**Published:** 2023-06-07

**Authors:** Frederick A. Hansen, Darren K. James, John P. Anderson, Christy S. Meredith, Andrew J. Dominguez, Nuttapon Pombubpa, Jason E. Stajich, Adriana L. Romero-Olivares, Shawn W. Salley, Nicole Pietrasiak

**Affiliations:** ^1^Department of Biology, New Mexico State University, Las Cruces, NM, United States; ^2^Jornada Experimental Range Department, New Mexico State University, Las Cruces, NM, United States; ^3^Montana Department of Environmental Quality, Helena, MT, United States; ^4^Plant and Environmental Sciences Department, New Mexico State University, Las Cruces, NM, United States; ^5^Department of Microbiology, Faculty of Science, Chulalongkorn University, Bangkok, Thailand; ^6^Department of Microbiology and Plant Pathology, University of California, Riverside, Riverside, CA, United States; ^7^U.S. Department of Agriculture-Natural Resources Conservation Service, Jornada Experimental Range, Las Cruces, NM, United States; ^8^School of Life Sciences, University of Nevada, Las Vegas, Las Vegas, NV, United States

**Keywords:** bacteria, cyanobacteria (blue-green algae), archaea, fungi, dryland, biocrust, topsoil, soil geomorphic template

## Abstract

**Introduction:**

Soil microbial communities, including biological soil crust microbiomes, play key roles in water, carbon and nitrogen cycling, biological weathering, and other nutrient releasing processes of desert ecosystems. However, our knowledge of microbial distribution patterns and ecological drivers is still poor, especially so for the Chihuahuan Desert.

**Methods:**

This project investigated the effects of trampling disturbance on surface soil microbiomes, explored community composition and structure, and related patterns to abiotic and biotic landscape characteristics within the Chihuahuan Desert biome. Composite soil samples were collected in disturbed and undisturbed areas of 15 long-term ecological research plots in the Jornada Basin, New Mexico. Microbial diversity of cross-domain microbial groups (total Bacteria, Cyanobacteria, Archaea, and Fungi) was obtained via DNA amplicon metabarcode sequencing. Sequence data were related to landscape characteristics including vegetation type, landforms, ecological site and state as well as soil properties including gravel content, soil texture, pH, and electrical conductivity.

**Results:**

Filamentous Cyanobacteria dominated the photoautotrophic community while Proteobacteria and Actinobacteria dominated among the heterotrophic bacteria. Thaumarchaeota were the most abundant Archaea and drought adapted taxa in Dothideomycetes and Agaricomycetes were most abundant fungi in the soil surface microbiomes. Apart from richness within Archaea (*p* = 0.0124), disturbed samples did not differ from undisturbed samples with respect to alpha diversity and community composition (*p* ≥ 0.05), possibly due to a lack of frequent or impactful disturbance. Vegetation type and landform showed differences in richness of Bacteria, Archaea, and Cyanobacteria but not in Fungi. Richness lacked strong relationships with soil variables. Landscape features including parent material, vegetation type, landform type, and ecological sites and states, exhibited stronger influence on relative abundances and microbial community composition than on alpha diversity, especially for Cyanobacteria and Fungi. Soil texture, moisture, pH, electrical conductivity, lichen cover, and perennial plant biomass correlated strongly with microbial community gradients detected in NMDS ordinations.

**Discussion:**

Our study provides first comprehensive insights into the relationships between landscape characteristics, associated soil properties, and cross-domain soil microbiomes in the Chihuahuan Desert. Our findings will inform land management and restoration efforts and aid in the understanding of processes such as desertification and state transitioning, which represent urgent ecological and economical challenges in drylands around the world.

## 1. Introduction

Drylands cover over 40% of earth’s terrestrial surface (Bowker et al., 2005) and are home to a variety of organisms that have adapted to survive in hot and dry conditions. Among these organisms are microbes living in a thin topsoil layer playing critical roles in the ecosystem. It is within this surface layer that we find greater values of organic matter and higher biological activity, mainly due to the greater abundance of soil microbes (Garcia-Pichel et al., 2003). However, our knowledge of what shapes their abundance, diversity, and distribution is still sparse.

One of the most established and beneficial surface soil microbial communities in drylands are found in biological soil crusts (biocrusts) which can locally occupy up to 80% of the ground (Rosentreter and Belnap, 2003) and are estimated to cover *circa* 12% of global terrestrial surfaces (Rodriguez-Caballero et al., 2018). Biocrusts are living soil aggregates that contain microbial communities with diverse evolutionary lineages including bacteria, cyanobacteria, archaea, eukaryotic algae, free living fungi, bryophytes, lichens, and associated microfaunal organisms (Hernandez and Knudsen, 2012; Büdel et al., 2016; Maier et al., 2018; Abed et al., 2019; Pombubpa et al., 2020; Omari et al., 2022). Biocrust microbes and other surface soil microbial communities contribute to key ecological functions in drylands including enhancing soil stability through aggregation by cyanobacterial exopolysaccharides and fungal proteins, which reduces erosion from wind and water (Belnap and Gillette, 1998; Wright and Upadhyaya, 1998; Eldridge and Leys, 2003; Pietrasiak et al., 2013). These communities also enhance nutrient cycling (Weber et al., 2015; Baumann et al., 2018; Crain et al., 2018) and bioweathering (Souza-Egipsy et al., 2004; Jung et al., 2020a), as well as contribute to nitrogen and carbon fixation from the atmosphere into the soil (Elbert et al., 2012). This influx of nitrogen and carbon into the soil has been shown to significantly contribute to plant essential nutrient uptake and availability in vascular plants that grow in soil with biocrust (Harper and Belnap, 2001). Biocrust have also been found to influence soil hydrology, increasing water infiltration while reducing runoff compared to areas without biocrust cover (Belnap et al., 2013).

Living in and on the topmost layer of soil, biocrust and other surface soil communities are exposed to abiotic factors and human disturbances, rendering them extremely vulnerable to climate change and impacts from overgrazing, trampling, and off-road vehicles. An extensive body of literature exists demonstrating disturbed areas have much lower biocrust abundance than undisturbed areas (Nash et al., 1977; Thomas and Dougill, 2006; Read et al., 2008; Concostrina-Zubiri et al., 2014; Ferrenberg et al., 2015). Areas with disturbed surface soil communities are at a greater risk of undergoing desertification and plant community transitions, which diminish land productivity from an agricultural perspective, consequently impacting the economy. For example, one study estimated the global costs of desertification to equal $23 billion/year in lost income and human displacement (Bowker et al., 2005). Full recovery of a disturbed biocrust area can take over 100 years (Briggs and Morgan, 2011). To mitigate further soil microbial disturbances and facilitate soil recovery in already disturbed areas via integrating the microbiome in restoration efforts, it is important to understand the composition of dryland soil microbiomes and identify factors influencing distribution of biocrust and other surface soil communities.

Although it has been recognized that soil microbes occurring in the plant interspaces are patchily distributed (Martirosayan et al., 2013), the abiotic and biotic factors affecting this distribution can be complex and greatly dependent on scale. Some studies have shown that soil pH and electrical conductivity (EC, a measure of soil salt content) can be major factors influencing biocrust composition, microbial growth, and diversity within desert ecosystems (Danin and Barbour, 1982; Johansen, 1993; Sand-Jensen and Jespersen, 2012; Stovall et al., 2022), while other studies argue that pH and EC have no relationship to biocrust development and diversity (Read et al., 2008; Ochoa-Hueso et al., 2011; Pietrasiak et al., 2011a). Several studies have related soil texture and moisture to biocrust abundance, finding that fine textured soils with high moisture content facilitate biocrust development (Read et al., 2008; Clark et al., 2009; Pietrasiak et al., 2011b). Biocrusts are also more prevalent in areas that have discontinuous vegetation, such as shrublands, where light can more easily reach the soil surface and fuel the photosynthetic processes of biocrust microbial communities (Mager and Thomas, 2011; Omari et al., 2022). Recently, geomorphology also was found to be influential (Pietrasiak et al., 2013, 2014a,b; Williams et al., 2013). Nonetheless, all these environmental factors have generally been analyzed treating biocrust as an undifferentiated whole, or responses have been investigated at the functional group level, while their impact on the particulars of microbial community composition and structure remains much less understood (Pombubpa et al., 2020).

In this study, our goal was to gain a comprehensive understanding of Chihuahuan Desert surface soil microbial communities and factors contributing to their distribution and abundance. The Chihuahuan Desert has been extensively studied at the Jornada Experimental Range since 1915 and within the Jornada Basin Long Term Ecological Research program since 1982. Seminal studies on cattle grazing dynamics, vegetation states and transitions, land potential, and the concept of the soil-geomorphic template were conducted here (Havstad et al., 2006; Bestelmeyer et al., 2015; Monger et al., 2015; Peters et al., 2015). However, surface soil microbial communities and their environmental drivers have not yet been thoroughly investigated. Thus, we characterized the distribution, composition, and structure of surface soil microbiomes found in five vegetation types of the Chihuahuan Desert and identified environmental factors shaping soil microbiome diversity. Environmental variables included vegetation types, landform types, soil properties, and trampling disturbance. We asked the questions: are vegetation characteristics more influential than soil or landscape characteristics? Are previously reported predictors of soil microbial diversity (such as pH, EC, and texture) similarly influential in the Chihuahuan Desert? How is disturbance shifting community composition and structure? Are specific taxonomic groups of microbes responding similarly, or do their sensitivity and resilience differ?

Based on the literature, we predicted that Jornada soil surface microbiomes would be spatially structured. Specifically, we predicted that vegetation type, as determined by dominant vegetation, would be the most influential variable explaining differences in microbial composition and alpha diversity, due to its effect of determining plant interspace size, connectivity, and extent, followed by geomorphology and soil properties. As cyanobacteria and archaea are more tolerant to salinity and alkalinity, we predicted that greater cyanobacteria and archaea diversity would be linked to high pH, high EC, and fine textured soils. Within fungi, we expected no strong relationships with specific soil properties and landscape features to emerge, due to the diverse life histories and dispersal strategies of this microbial group. As we focused on surface soil microbial communities, we also predicted that areas disturbed by occasional trampling would have lower soil microbial diversity and altered community structure, compared to otherwise similar soils without trampling impacts. To investigate these relationships, composite soil samples were systematically collected from disturbed and undisturbed areas within 15 long-term plots in the Jornada Basin of Southern New Mexico. Insights gained on dryland soil microbiomes are not only needed to understand past and present soil ecology of the Chihuahuan Desert but will also be vital in preserving or rehabilitating these and other desert ecosystems impacted by land use changes, recreation, and development.

## 2. Materials and methods

### 2.1. Study sites and sampling methods

**Figure 1:**
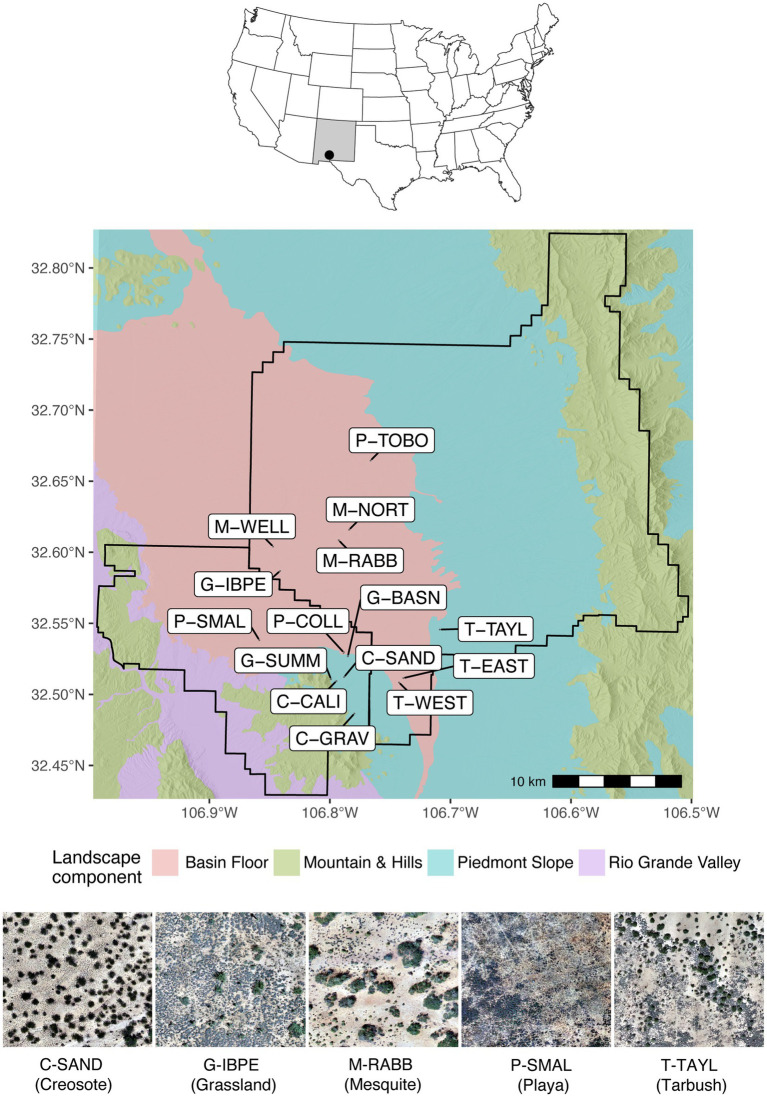
Location of the Jornada Basin in southern New Mexico, United States (top), the four major landscape components with 15 sites included in the study (center), and aerial imagery from representative sites for each vegetation zone studied including visible trampled walking paths (bottom). Site labels reflect vegetation zone components including creosote: C-CALI, C-GRAV, and C-SAND, grassland: G-BASN, G-IBPE, and G-SUMM, mesquite: M-NORT, M-RABB, and M-WELL, playa and bottomland: P-COLL, P-SMAL, and P-TOBO, and tarbush: T-EAST, T-TAYL, and T-WEST. Boundaries shown within the Jornada Basin are the Chihuahuan Desert Rangeland Research Center (lower left) and USDA-ARS Jornada Experimental Range (center and upper right). Aerial imagery was modified after Wojcikiewicz and Hanan (2022).

This study was performed at the Jornada Basin, located in the Chihuahuan Desert of Southern New Mexico (Figure 1). In association with the USDA-ARS Jornada Experimental Range and the NMSU Chihuahuan Desert Rangeland Research Center, the Jornada Basin Long Term Ecological Research program established 15 long-term monitoring grazing exclosures in 1982 that represent five dominant vegetation types within the basin, with three representative sites varying in vegetation density for each vegetation type (Figure 1).

Except for one playa site, each site is characterized by a 7 × 7 grid composed of 10 m × 10 m monitoring squares covering an area of 4,900 m^2^ per site. Each long-term site is either protected from cattle by being located within fenced pastures excluded from grazing or is protected as a smaller exclosure by its own barbed-wire fence. Researchers investigating dryland net primary production (NPP) visit the sites at least three times a year to take NPP measurements. The researchers hereby follow specific walking paths within the plots to prevent trampling disturbance elsewhere. Additionally, this path is also visited partially once per month for soil moisture determination. These walking paths have been compacted due to this long-term regular visitation and were considered the disturbed areas in our study.

To characterize the microbial communities of these 15 NPP sites, we separately collected and composited soil samples from undisturbed and disturbed areas within each NPP site, totaling 30 composite soil samples. Soil samples were taken between June 16 and July 6 of 2016 in a systematic pattern that represented each entire NPP site, using a 1 cm deep × 6 cm wide brass soil core in 24 of the 49 monitoring squares (skipping every other square). For undisturbed samples, we took three standardized steps (ca 1.5 m) on the walking path toward a monitoring square, and then three steps to the left and sampled directly at our feet. This technique allowed for a non-biased systematic sample collection, especially in instances where well-developed but patchy biocrusts were present. Disturbed samples were taken at three steps on the walking path that researchers would use quarterly to measure NPP in each square assigned to be sampled. Surface soils were sampled in dry conditions during microbial dormancy and equipment was cleaned using 95% ethanol between each soil core collection. Samples were stored in a cooler with ice packs and then transported to New Mexico State University, where they were placed in a 4°C cold room until laboratory sample processing, which occurred within 2 weeks of sample collection.

When soil sampling occurred, 14 of the 15 sites had access to weather stations and soil moisture probes with data available online (Anderson, 2022a,b,c,d,e,f,g,h,i,j,k,l,m,n,o; Duniway, 2022a,b,c,d,e,f,g,h,i,j,k,l,m,n,o). Supplementary Table 2 lists maximum daily air temperature, antecedent rainfall, and average soil moisture content at 10 cm depth for each of the 14 available sites at the time of soil sampling.

### 2.2. Physical and chemical soil properties

In the laboratory, the 30 composite samples were weighed to determine their total mass. Using sterile techniques to minimize cross-contamination between samples, we separated gravel and large plant litter pieces from the fine earth fraction using a 2 mm metal sieve. Soil aggregates were crushed and included in the fine earth fraction. Percent gravel content was determined gravimetrically. A well-mixed sub-sample of the fine earth fraction was taken using sterile technique and stored in a − 80°C freezer for later DNA-based procedures.

We then assessed soil pH, electrical conductivity (EC), texture, and sand fractions of each composite soil sample. We determined pH and EC using a well-mixed 100-g subsample of each composite sample. The subsamples were placed in a sealable container and slowly wetted until they became a saturated paste. The containers were then closed, and the paste was allowed to sit for a minimum of 4 h. After the waiting period, an OAKLON pH/CON 510 Series pH probe was inserted into the paste and its measurement was recorded. To measure EC, the soil solution from the saturated paste was extracted using a vacuum system. We then used an Accumet pH meter 50 EC probe to measure the EC of each extract. Soil texture was determined by a combination of hydrometer determination and wet sieving. For coarse textured sites (creosote, mesquite, tarbush, and grass sites) 100 g of well-mixed soil were used. For the finer textured playa sites, only 50 g of soil were used. We determined clay content after 24 h following the hydrometer methodology of Gee and Or (2001). After taking hydrometer readings, the suspension was sieved using a 53 μm pore sized sieve to obtain the percent sand fraction gravimetrically, by thoroughly rinsing the subsample using deionized water to flush silt and clay particles. The sand remaining on the sieve was then transferred into a beaker, dried in a 105°C oven for 2 days, packaged in Whirl-pak bags and sent to the Environmental Analytical Lab at Brigham Young University, Utah for sand fraction analysis.

### 2.3. Landscape features

We investigated the following landscape features for their relationships with microbial diversity and structure: vegetation type, perennial plant biomass, lichen biocrust cover, geomorphology (landscape components and landforms), soil parent material, ecological site, and ecological state (Supplementary Table 1). Vegetation type classification (referred to as vegetation zone hereafter) using the dominant plant community at each site was scored as Creosote, Mesquite, Grassland, Tarbush, or Playa. To investigate the influence of plant biomass we utilize the perennial grass and shrub annual aboveground NPP for each NPP site recorded in 2016 by Peters and Huenneke (2022) (Supplementary Table 2). As part of long-term vegetation monitoring, 10 line-point intercept points are collected at each of the 49 long-term quadrats at each site for a total of 490 points per site for soil surface characterization. We used the soil surface data from the spring 2016 monitoring to estimate lichen crust cover at each of the sites. Data appear in Supplementary Table 2.

Following the Natural Resources Conservation Service Geomorphic Description System (Schoeneberger and Wysocki, 2017), we assigned each site a landscape and landform category. Landscapes represent broad assemblages of spatially associated features while landforms depict discrete earth surface features at a finer scale. In our study, “Landscape” was identified and scored either as Piedmont Slope or Basin Floor, while “Landform” included Playa, Alluvial Flat, Alluvial Plain, and Fan Piedmont (Supplementary Table 1). Monger et al. (2006) was used to assign soil parent material. Ecological site and ecological state designations (Steele et al., 2012) were based on the USDA Jornada Experimental Range ecological site and state map (Burkett and Bestelmeyer, 2022). Ecological sites are classes of land defined by recurring soil, landform, geological, and climate characteristics. Ecological site class concepts differ from plant community classification in that they describe the ecological potential of land areas based on plant production, plant species composition, and dynamic soil properties at reference conditions, ecosystem services provided, response to management, and processes of degradation and restoration (Duniway et al., 2010). Ecological sites identified for each NPP site included Playa, Bottomland, Loamy, Sandy, Gravelly Sand, and Gravelly (Supplementary Table 1). Ecological sites can be observed in one or more alternative ecological states, which are typically distinguished by their plant community characteristics (Steele et al., 2012). States assigned to the 15 NPP sites were Reference Grassland, Altered Grassland, Exotic Invaded, Shrub-Invaded, Shrubland, Shrubland with Exotic Grasses (Supplementary Table 1). Supplementary Table 1 describes study site classifications in more detail and Supplementary Table 2 lists the climate and plant biomass data used in our investigation.

### 2.4. Microbial community characteristics

To characterize the bacterial, cyanobacterial, archaeal, and fungal communities in our soils, we performed DNA extraction and next generation MiSeq sequencing. Genomic DNA was extracted from our soil samples using the DNeasy PowerLyzer PowerSoil Kit by QIAGEN (Hilden, Germany). We followed the manufacturer’s protocol with two modifications that enabled higher DNA yields from our samples: (1) the soil contained in each bead beating tube was homogenized at 4,500 rpm for 45 s using a Precellys 24 high-throughput homogenizer (Bertin Technologies, Montigny-le-Bretonneux, France); and (2) for the final step the elution buffer was allowed to sit on the filter for 5 min before final centrifugation. DNA extracts were stored in a −20°C freezer until amplicon metabarcoding via PCR.

We ran separate PCRs for the 16S rRNA gene marker to amplify bacterial (including cyanobacterial) and archaeal sequences, versus the ITS1 rRNA gene marker to amplify fungal sequences. PCR was performed using standard primer sets and protocols from the Earth Microbiome Project protocols (Caporaso et al., 2012; Smith and Peay, 2014). We amplified three replicates per sample and PCR cleanup was performed on the pooled PCR reactions for each sample, using the NucleoSpin Gel and PCR Clean-up kit from Macherey Nagel (Düren, Germany). Concentrations of the cleaned PCR products were then determined using an IMPLEN Nanophotometer machine (Westlake Village, CA, United States). Based on the final concentrations, PCR products were pooled in equimolar amounts for sequencing. The libraries for 16S and ITS amplified samples were sequenced on Illumina MiSeq in 2 × 300 PE base format at the University of California Riverside Core Genomic Sequencing Facility. Bioinformatics.

Raw sequencing data were processed by the New Mexico State University high performance computing cluster, “Discovery” (Trecakov and Von Wolff 2021). The program AMPtk (Palmer et al., 2018) merged forward and reverse raw sequence reads, grouped them by sample, removed primer sequences, trimmed sequence reads, removed chimeras, and created Amplicon Sequence Variants (ASVs). The ASVs were classified with a 99% similarity threshold using the AMPtk command: UNOISE3. Next, taxonomic classification was assigned to the ASVs with the program QIIME2 (Bolyen et al., 2019). The QIIME2 command: q2-feature-classifier, assigned taxonomic rank to bacterial, cyanobacterial, and archaeal ASVs using SILVA database version 132 (Quast et al., 2013). SILVA based classification of photoautotrophic cyanobacterial ASVs was then updated using a combination of BLASTN comparisons, phylogenetic placement in CYDRASYL (Roush et al., 2021), and the modern cyanobacterial taxonomic system per Komárek et al. (2014). Taxonomic ranking of fungal ASVs was completed in AMPtk, comparing sequence reads with existing fungal reads in the UNITE database (v1.4.3; Nilsson et al., 2019). The output files (ASV tables, taxonomy tables, and mapping files) were imported to R version 4.2.2 for statistical analysis.

### 2.5. Statistical analysis

We performed data processing, analysis, and visualization with R version 4.2.2 (Wickham et al., 2019; R Core Team, 2021). We conducted paired *t*-tests to check for disturbance effects for each soil chemical/physical response (percent gravel, percent sand, percent clay, EC, and pH), pairing the disturbed and undisturbed sample at each site.

To analyze the ASV data we relied primarily on the *phyloseq* package (McMurdie and Holmes, 2013). Preprocessing included removing low count ASVs and rarefactions. We rarefied our microbial datasets ensuring all samples have the same library size and minimizing sequencing depth differences that could otherwise confound data analysis (Gotelli and Colwell, 2001). After pruning ASVs with two or fewer counts, read counts from the ITS fungal sequences ranged from 18,619 to 18,768. Read counts from the 16S sequences ranged from 32,289 to 88,495 after we pruned ASVs with five or fewer counts. Samples were then rarefied to 32,289 reads. Because the 16S sequencing included both bacteria and archaea, after rarefying we created separate subsets for each of both domains. We further created a cyanobacteria subset from the total bacteria, resulting in four final subsets for our analyses: (1) total bacteria, (2) cyanobacteria, (3) archaea, and (4) fungi. We conducted data analyses separately for each subset.

For each sample, we calculated alpha diversity measures of observed richness, Chao1 diversity, and Simpson diversity, and then used linear models to test for differences between disturbed and undisturbed samples. Because we found no evidence of a disturbance effect, except for observed richness in Archaea (see Results), we used only the undisturbed samples (except for fungi at T-TAYL due to sequence failure in this one sample) in linear models to examine the effects of Vegetation Zone, Landscape, Landform, and Ecological Site on observed richness and on relative abundance of selected taxonomic groups. When three or more levels of an effect were present, we used the Tukey method to compare means. Residuals did not differ substantially from normality in any of the linear models. We used corrected Akaike information criterion (AICc, Mazerolle, 2020) to examine variable importance for observed ASV richness.

Polynomial regressions were used to examine the possible relationships between observed ASV richness and numeric soil variables (percent gravel, percent sand, percent clay, EC, and pH) as well as with vegetation biomass (perennial grass and shrub annual aboveground NPP in the year that sampling occurred). We used both disturbed and undisturbed samples and tested for significance of linear, quadratic and cubic effects. However, since low sample size limited predictive inference of polynomial regression, our goal was to suggest possible heuristic trends over the range of environmental variables observed.

To test for a disturbance effect in community composition, we used a permutational multivariate ANOVA (PERMANOVA) employing Bray–Curtis dissimilarity with the adonis function from the *vegan* package (Lahti and Shetty, 2017; Oksanen et al., 2020). The dependent variables were the matrix of relative abundance of each ASV, and we did not combine ASVs to any taxa level. However, we used the *microbiome* package (Lahti and Shetty, 2017) to apply a compositional transformation to ASV abundances. We also applied PERMANOVA on the undisturbed samples only, to examine the effects on species turnover of Vegetation Zone, Landscape, Landform, Parent Material, Ecological Site, and Ecological State. To determine how environmental variables influenced patterns in community composition we performed an environmental fit using both disturbed and undisturbed samples and included the same environmental variables as used in the polynomial regression analysis as well as maximum daily air temperature, soil moisture, and lichen crust cover. Significant correlations of environmental variables (=environmental fits) were overlaid and visualized as vectors in the NMDS ordination.

## 3. Results

### 3.1. Soil characterization

Paired *t*-tests revealed no differences between disturbed and undisturbed samples for all soil chemical and physical properties investigated in this study (*p* > 0.05 for all variables). However, soil properties varied largely in particle size and soil chemistry between the 15 Jornada Basin NPP sites. Gravel content of soil samples ranged from 0% at multiple playa sites to 38% at a creosote site (Table 1) with an overall average of 8%. Sand content varied most among the sites ranging from 2% at a playa site to 89% at a mesquite site. Playa sites were characterized by finer textures (loam to silty clay). Tarbush, creosote, grassland, and mesquite sites generally exhibiting coarser-sandier texture, with more loam at tarbush sites and higher sand fraction at the creosote (sandy loam), grassland (sandy loam or loamy sand), and mesquite sites (loamy sand). The pH of our sites ranged from 6.4 at a mesquite site to 7.5 at a playa site (Table 1). The EC at our sites ranged from 0.316 μs/cm at a mesquite site to 1.04 μs/cm at a tarbush site (Table 1).

**Table 1:** Basic soil physical and chemical characteristics for the 15 long term NPP sites, Jornada Basin, southern New Mexico, United States (data from undisturbed samples only).

Site	Gravel (%)	Sand (%)	Silt (%)	Clay (%)	pH	Electrical conductivity (μs/cm)
C-CALI	25.44	72.10	14.53	13.37	7.61	0.52
C-GRAV	33.95	65.80	23.75	10.45	7.17	0.77
C-SAND	17.67	74.80	16.86	8.34	6.77	0.47
G-BASN	3.10	74.30	13.23	12.47	7.15	0.53
G-IBPE	0.48	81.62	10.96	7.42	6.78	0.49
G-SUMM	38.44	78.60	13.06	8.34	6.94	0.63
M-NORT	1.08	83.20	6.47	10.33	6.76	0.37
M-RABB	0.68	88.70	3.98	7.32	6.44	0.55
M-WELL	2.46	82.90	8.83	8.27	6.89	0.44
P-COLL	0.05	5.40	54.78	39.82	7.50	0.98
P-SMAL	0.00	2.00	53.93	44.07	7.05	1.13
P-TOBO	0.00	42.69	38.75	18.56	7.23	0.72
T-EAST	0.09	63.10	23.62	13.28	7.16	0.67
T-TAYL	1.64	50.80	34.62	14.58	7.14	0.60
T-WEST	0.01	44.00	40.51	15.49	7.15	0.70

### 3.2. Taxonomic composition of the soil surface microbes

**Figure 2:**
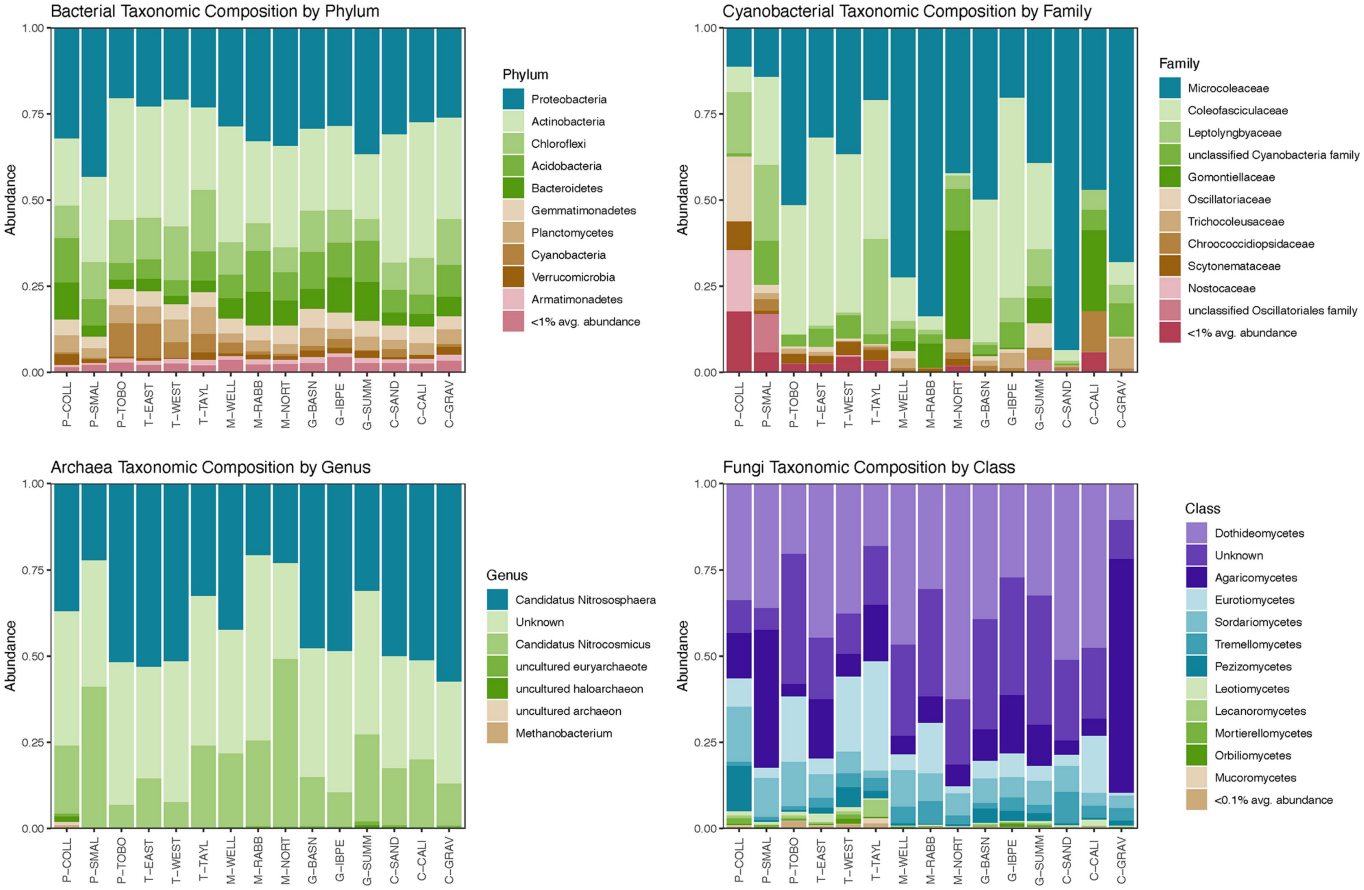
Taxonomic compositions of domain Bacteria, bacteria phylum Cyanobacteria (photoautotrophic only), domain Archaea, and phylum Fungi within domain Eukarya at the 15 long term NPP plots, Jornada Basin, New Mexico, United States. Plots show data from undisturbed samples. Numeric values of relative abundances appear in Supplementary Table 3.

**Figure 3:**
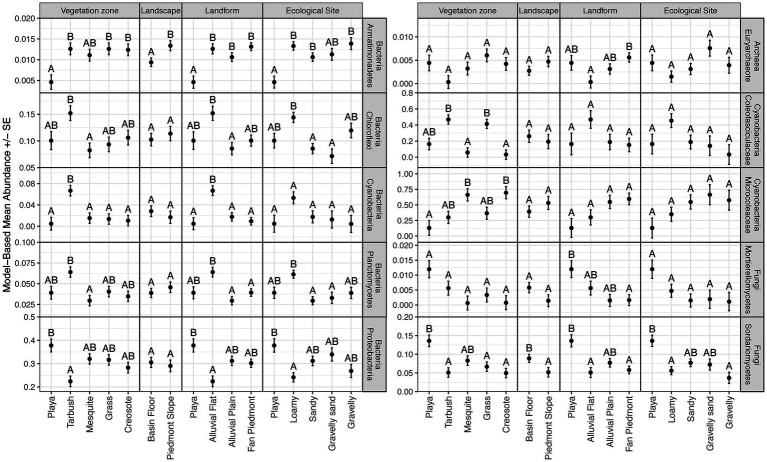
Model-based means and standard errors of relative abundances for taxa in Bacteria, Archaea, Cyanobacteria, and Fungi with significant differences. Within a panel, means with the same letter are not different at α = 0.05.

For domain Bacteria, the taxonomic composition at the phylum level was characterized by high abundances of Proteobacteria (average relative abundance 29.3%), Actinobacteria (average relative abundance 28.8%), Chloroflexi (average relative abundance 10.8%), Acidobacteria (average relative abundance 8.3%), and Bacteroidetes (average relative abundance 6.0%) for all sites (Figure 2; Supplementary Table 3). These five phyla comprised 75.8–89.7% of the bacterial microbiomes at the 15 NPP sites surveyed (Supplementary Table 3). Significant differences in relative abundances of bacterial phyla across landscape features were found in the Armatimonadetes, Chloroflexi, Cyanobacteria, Planctomycetes, and Proteobacteria phyla (Figure 3; Supplementary Table 8). In general, Armatimonadetes, Chloroflexi, Cyanobacteria, and Planctomycetes showed similar results in the linear models with the highest relative abundance in Tarbush vegetation and Alluvial Flats and generally low abundances in Playas (Figure 3; Supplementary Table 8). Across the top 10 bacterial phyla, we found only for Armatimonadetes a significant difference in Landscape category with higher relative abundances on the piedmont slope. Abundances of Proteobacteria responded distinctly different from the other bacterial phyla and were highest in the Playas while lowest abundance were detected in Tarbush sites and Alluvial Flats which were associated with Loamy Ecological sites (Figure 3). Further, relative abundance differences were distinct at finer taxonomic levels within individual microbial phyla (See Archaea, Cyanobacteria, and Fungi in Figure 2 and Supplementary Figure 1). We also detected a large proportion of unclassifiable ASVs in Cyanobacteria, Thaumarchaeota, and Fungi.

Cyanobacterial abundances varied greatly from being a rare microbial community component (<0.001% relative abundance in domain Bacteria) for P-COLL, P-SMAL, M-RABB, M-NORT, G-SUMM, C-CALI, and C-GRAV to 5–10% relative abundance at P-TOBO, T-EAST, T-WEST, and T-TAYL (Supplementary Table 4). When contrasting landscape features, Cyanobacteria were highest in Tarbush vegetation and Alluvial Flat landforms (Figure 3). Cyanobacteria were dominated by Oscillatoriales taxa (average 78.1% relative abundance) with ASVs classifying to *Microcoleus* being the dominant genus (average relative abundance 44.2%), while others were locally co-dominant such as *Allocoleopsis* (P-SMAL, G-BASN, and G-IBPE), *Crinalium* (M-NORT, M-RABB, and C-CALI), *Pycnacronema* (G-SUMM), and *Parifilum* (G-BASN; Supplementary Table 5). An interesting contrast in relative abundance responses to landscape features were detected for Coleofasciculaceaen taxa (e.g., *Pycnacronema*, *Parafilum*, and *Allocoleopsis*) with highest abundances in Tarbush and Grassland compared to Microcoleacean taxa (*Microcoleus*) where significantly higher abundances were detected for Mesquite and Creosote vegetation (Figure 3; Supplementary Table 9). The second most abundant cyanobacterial order was Synechococcales, averaging 10.9% relative abundance and ranging from 1.3 to 31.0% in Cyanobacteria abundances across the 15 sites (Figure 2; Supplementary Table 5). The most abundant genera within Synechococcales were *Phormidesmis* and an unclassified Leptolyngbyaceae genus. Nostocales (3.1%) and Chroococcidiopsidales (1.5%) represented only minor components at the 15 NPP sites overall but were sometimes locally abundant with Nostocales being abundant at P-COLL (32% with *Scytonema* and an unclassified Nostocacean genus) and Chroococcidiopsidales at C-CALI (11.8% of an unclassified Chroococcidiopsidaceae genus). Members of the Chroococcales order were recorded at the 15 NPP sites but were rare. An unclassified cyanobacterial order was identified as being locally abundant at P-SMAL and M-NORT (Supplementary Table 5).

For domain Archaea, the relative abundance at the phylum level was distinctly dominated by Thaumarchaeota in all 15 NPP sites (>99% relative abundance; Figure 2; Supplementary Table 6). An average of 60% relative abundance within Thaumarchaeota was composed by Candidatus *Nitrososphaera* and Candidatus *Nitrosocosmicus*, two ammonia oxidizing archaea. The remaining portion was represented by an unclassified Thaumarchaeota genus. Euryarchaeota and Nanoarchaeaota were rare (<1% abundance) and ASV’s recorded were mostly unclassified archaea (Supplementary Table 6). At P-COLL Euryarchaeota reached an abundance of 4% with *Methanobacterium* (0.88%) being the only classifiable genus of this archaea phylum (Supplementary Table 6). However, an uncultured Euryarchaeota genus was the only Archaean group revealing significant differences in landscape structure in the linear models (Supplementary Table 10). For this group, abundances were lowest in Alluvial Flats and highest in Fan Piedmonts (Figure 3; Supplementary Table 10).

The fungal community was dominated by Ascomycota (average relative abundance of 68.8%) and to a lesser degree by Basidiomycota (average relative abundance of 19.3%, Supplementary Figure 1; Supplementary Table 7). Ascomycota taxa were highly abundant (>75%) in P-COLL, T-WEST, all mesquite sites, C-SAND and C-CALI, but not at C-GRAV where taxa in Basidiomycota dominated instead. An unclassified fungal phylum occurred at 10.5% average relative abundance. Mortierellomycota, Rozellomycota, and Mucoromycota were recorded at the 15 NPP sites but averaged <1% relative abundance. Mortierellomycota reached >1% at P-COLL and T-WEST, while Rozellomycota reached 1.2% at T-WEST and G-BASN and Mucoromycota 1.4% at T-TAYL (Supplementary Table 7). Within fungi, relative abundances only differed significantly across landscape features for the classes Mortierellomycetes and Sodariomycetes (Figure 3; Supplementary Table 11). Among the Mortierellomycetes, only Landform was important to explain differences in relative abundances, with Playas having the highest and Fan Piedmont having the lowest relative abundances. Sodariomycete taxa showed abundance differences by Vegetation, Landscape, Landform, and Ecological Site. Like Mortierellomycetes abundances were highest in Playas and lowest in Fan Piedmonts. This pattern was supported by relative abundance being highest in Playa vegetation, Basin Floor landscape, and Playa ecological sites while being lowest on Grassland, Tarbush, and Creosote as well as Loamy and Gravelly ecological sites (Figure 3; Supplementary Table 11).

At finer taxonomic resolution additional interesting patterns emerged within fungi. The most abundant class within Ascomycota were Dothideomycetes (average relative abundance of 36.0%) while Agaricomycetes dominated the Basidiomycota (average relative abundance of 15.4%, Figure 2; Supplementary Table 7). Several families in Pleosporales showed differences in local abundance including Didymellaceae (average 8.6%, highest abundance in M-WELL and C-CALI), Sporomiaceae (average 5.7%, highest abundance in G-BASN and C-SAND), and Coniothyriaceae (average 1.1%, highest abundances in tarbush sites). An unknown family (52.0%, nearly all sites with >20% relative abundance) comprised the Dothideomycetes dominance (Supplementary Figure 1; Supplementary Table 7). In Agaricomycetes, the families Agaricaceae (4.8%) and Nidulariaceae (2.6%) were most abundant (Supplementary Table 7). However, their abundances were mainly determined by local high abundance values in one or two sites only, with Nidulariaceae reaching 38.3% in P-SMAL and Agaricaceae reaching 60.6% in C-GRAV and 9.3% in G-IBPE (Supplementary Table 7). Yeast-like fungi such as taxa in Filobasidiaceae (Tremellomycetes, Basidiomycota) and Aureobasidiaceae (Dothideomycete, Ascomycota) were rare but occurred at abundances of 1–6% in at least half of the Jornada Basin NPP sites (Supplementary Figure 1; Supplementary Table 7).

### 3.3. Factors influencing microbial taxon richness

**Figure 4:**
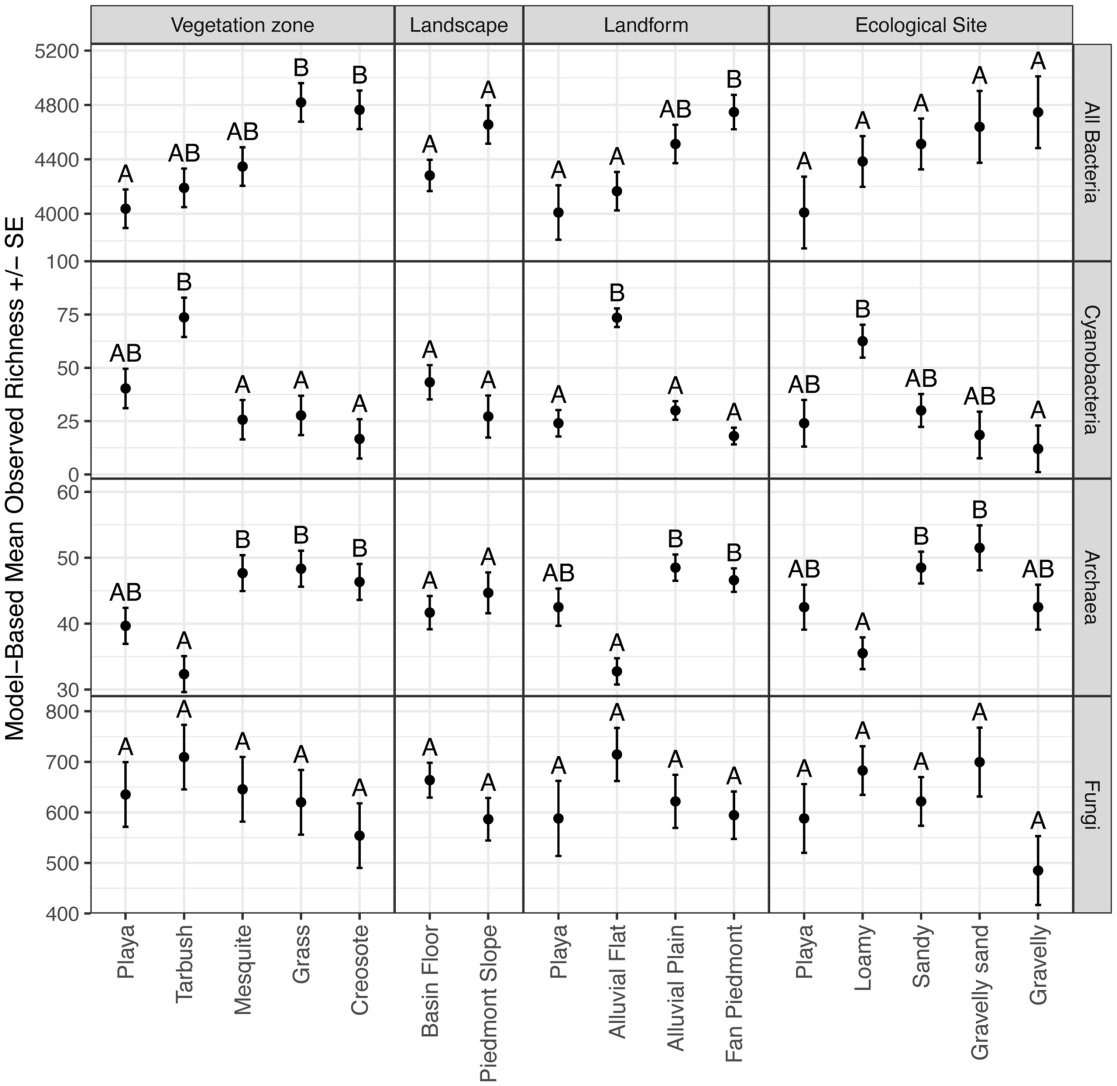
Model-based means and standard errors of observed species richness. Within a panel, means with the same letter are not different at α = 0.05.

Our hypothesis that disturbance was an influential factor in microbial diversity was generally unsupported by our data, as we found no evidence of differences between disturbed and undisturbed samples for total Bacteria, Cyanobacteria, and Fungi, across all three analyzed diversity indices (observed richness, Chao diversity, Simpson diversity; *p* ≥ 0.05). Archaea showed higher observed richness in undisturbed samples (difference of 2.80 ± 0.98 S.E., *p* = 0.0124) but no differences with respect to Chao and Simpson diversity (*p* = 0.290 and 0.310 respectively). Among landscape features, differences were detected with vegetation zone and landform for Bacteria; with vegetation zone, landform, and ecological site for Cyanobacteria and Archaea; while no differences were observed for Fungi (Figure 4). Total Bacteria richness was highest in grassland and creosote sites, which were mostly located on the fan piedmont, while it remained lowest in playa sites. Interestingly, tarbush sites were highest in Cyanobacteria richness versus lowest in Archaea richness (Figure 4). This pattern was also detected for alluvial flat landform sites (T-East and T-West) and loamy ecological sites (all three tarbush sites, Figure 4). Ecological state and parent material was not tested, due to unequal sample numbers in each category.

Akaike information criterion analyses included all landscape feature categories. For Bacteria and Fungi richness, no strong predictors were detectable, i.e., the null model was the best model. In contrast, landform was the most important predictor for Cyanobacteria and Archaea richness (100 and 98.51% of AICc weight respectively, Supplementary Table 3).

Polynomial regression related continuous soil data to microbial richness (Supplementary Figure 2). In general, we could observe first gradients and patterns in the data, but sample size was too limited, and more data are needed to characterize these fully. Total Bacteria had linear relationships for % clay and perennial grass biomass as well as cubic relationships for % gravel, % sand, and EC. Cyanobacteria showed linear relationships for % Gravel, quadratic for % Clay, EC, pH, and cubic for % Sand. Archaea was linear for EC, cubic for % gravel and pH and quadratic for soil texture. In Fungi, there were no discernible patterns.

### 3.4. Factors influencing community composition and species turnover

Disturbance was not a significant predictor of changes in community composition for Bacteria, Cyanobacteria, Archaea, and Fungi communities (PERMANOVA, *p* = 1.00, 1.00, 0.97, 1.00). We then tested influences of all six landscape features (vegetation zone, landscape component, landform, parent material, ecological site and state) on community composition and species turnover for the four microbial subsets. Compared to alpha diversity, species turnover (beta diversity) was more strongly influenced by these variables, i.e., almost all variables were significant, and they explained a larger portion of the variability in the compositional data (Table 2). In Bacteria, Cyanobacteria, Archaea, and Fungi communities the highest proportions of variation in composition were explained by ecological site (PERMANOVA, *p* = 0.01, 0.02, 0.03, 0.01) and by ecological state (PERMANOVA, *p* = 0.01, 0.02, 0.03, 0.01). Ecological site was most important for Bacteria and Fungi, while ecological state was the most important for Cyanobacteria and Archaea. Vegetation zone and landform explained 32–48% of the variability and were the next most important predictors. Parent material was usually less important in predicting dissimilarities among microbes but was still significant for each subset except Archaea. Landscape was the only landscape feature not found to be important for any subset (Table 2).

**Table 2:** Permutational multivariate ANOVA (PERMANOVA) results for community composition analyses.

Term	Total bacteria		Cyanobacteria		Archaea		Fungi^+^	
	*R* ^2^	*p*-value	*R* ^2^	*p*-value	*R* ^2^	*p*-value	*R* ^2^	*p*-value
Disturbance	0.01	1.00	0.01	1.00	0.01	0.97	0.02	1.00
Ecological site	**0.54**	**0.01**	**0.51**	**0.02**	**0.51**	**0.03**	**0.47**	**0.01**
Ecological state	**0.47**	**0.03**	**0.60**	**0.01**	**0.53**	**0.03**	**0.42**	**0.02**
Landscape	0.07	0.34	0.06	0.51	0.02	0.96	0.08	0.23
Landform	**0.46**	**0.01**	**0.48**	**0.01**	**0.36**	**0.05**	**0.32**	**0.01**
Soil parent material	**0.42**	**0.01**	**0.40**	**0.04**	0.33	0.25	**0.38**	**0.01**
Vegetation zone	**0.46**	**0.01**	**0.48**	**0.01**	0.44	0.06	**0.40**	**0.01**

*p*-values were determined from 99 unique permutations of the data. ^+^No undisturbed sample was available from T-TAYL; used disturbed sample as substitute.Bold values indicates cases where R2 is significantly different from zero at α = 0.05.

**Figure 5:**
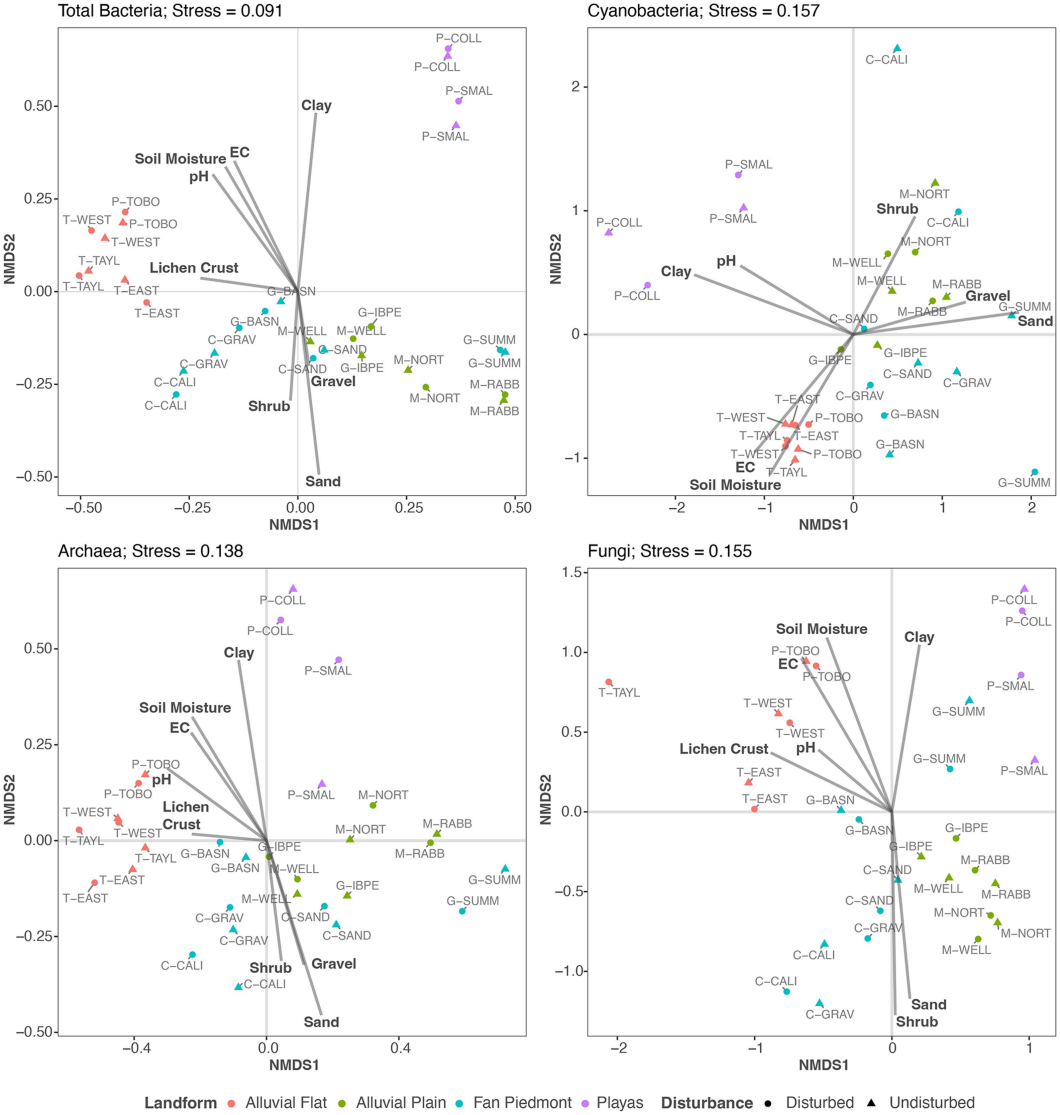
NMDS ordinations of Total Bacteria, Cyanobacteria, Archaea, and Fungi with overlaid environmental fit results of soil properties, lichen biocrust cover, and perennial plant biomass data. Only significant environmental variables (*p* < 0.10) are shown as vectors.

All four NMDS ordination models obtained stress values <0.2 and revealed three distinct sample point clusters (Figure 5), suggesting that similar drivers shape similarities and dissimilarities of microbial community composition for all four microbial groups. Landform best described this clustering across the 15 samples (Figure 4). Samples obtained from Playa and Alluvial Flat landforms distinctly grouped as two separate clusters, while Alluvial Plain and Fan Piedmont samples formed a large overlapping sample point cluster (Figure 5). Next, we examined how the continuous soil, air temperature, lichen crust cover, and vegetation biomass data influenced compositional clustering patterns of our soil surface communities, using an environmental fit analysis with the two first NMDS ordination axes (Figure 5; Supplementary Table 12). Gravel, soil, texture (%clay and sand), soil moisture, EC, pH, lichen crust cover, and shrub biomass had significant relationships with the NMDS ordination axis scores (Figure 5; Supplementary Table 12). For all four microbial groups, similar relationships explained observed clustering patterns: fine textured soils high in EC (indicating high salt content) and/or pH values and with soil moisture values of 5–20% (Supplementary Tables 2, 12) characterized alluvial flat and playa microbial communities, while coarse textured and gravelly soils associated with the greatest shrub biomass and lowest soil moisture (<5%, Supplementary Tables 2, 12) related to alluvial plain and fan piedmont communities (Figure 5). Lichen crust cover was important in associating with Total Bacteria, Archaea, and Fungal community dissimilarities but not Cyanobacteria (Figure 5; Supplementary Table 12). Interestingly, the association of plant biomass was much weaker in Cyanobacteria but associated more strongly with axis 2 in Fungi (Figure 5).

## 4. Discussion

In this study, we characterized the soil microbial communities including total Bacteria, Cyanobacteria, Archaea, and Fungi within diverse vegetation zones and landscape features of the Chihuahuan Desert, to identify important factors influencing these communities. Our study confirms that dryland soil microbiomes are unique, with significant diversity of unknown taxa, including some that can occur locally in high abundances. We also present novel relationships between dryland landscape features and the soil surface microbiome that enhance our understanding of their composition and structure in these systems.

### 4.1. Unique and understudied microbial composition exists in Chihuahuan Desert soils

We revealed Chihuahuan Desert soil microbial communities to be unique, diverse, and poorly studied. This became apparent when we explored microbial diversity at finer taxonomic resolution, e.g., in Cyanobacteria, domain Bacteria; in Thaumarchaeota, domain Archaea, and in Fungi, domain Eukarya. All microbial groups were composed of typical dryland adapted taxa but also showed many unclassifiable ASVs from species to order. While we have little to no knowledge of their basic biology and roles in the dryland ecosystems, the information presented here will help guide priorities for taxonomic descriptions of organisms most likely to be highly unusual compared to known taxa, and/or relatively abundant thus implying potential importance to local or regional ecosystems.

Although Cyanobacteria averaged only 3% of the relative abundance in the domain Bacteria across the 15 NPP sites, they were richest and reached 5–10% relative abundance values at P-TOBO, T-EAST, T-WEST, and T-TAYL. Although these abundance values are relatively low compared to other values reported in the literature (e.g., in average *ca.* 40% in Mojave biocrust in Pombubpa et al., 2020, max of *ca.* 50% in Steven et al., 2013, and max of 35% in Abed et al., 2019) they fall within reported abundance ranges of 3–50% for desert top soils (Steven et al., 2013; Maier et al., 2018; Abed et al., 2019). Our observed values also reflect the broad gradient of biocrust established at the Jornada Basin from no or little crust to distinct land surface covers. Within-site biocrust patchiness further led to a reduction of the cyanobacteria component within our composite samples (Figure 1). However, the four sites with highest cyanobacteria abundances (P-TOBO, T-EAST, T-WEST, and T-TAYL) had visually the most distinct lichen and dark cyanobacterial crust cover of all the 15 NPP sites. They also clustered tightly together in the NMDS ordination plots for all four microbial groups investigated and correlated with high lichen cover in the environmental fit (Figure 5), all suggesting that presence and composition of biocrust could be highly influential in shaping the soil surface microbiomes at those sites (supporting findings by Omari et al., 2022). Interestingly, richness in Total Bacteria was lowest for these biocrusted sites, while it was high in fan piedmont sites that had low to no biocrust presence (Figures 2, 3). Lower bacterial richness in well-developed biocrusted soils compared to non-crusted soils also was detected in the Arabian, Western Australian, and the North American Mojave Deserts (Abed et al., 2019; Moreira-Grez et al., 2019; Pombubpa et al., 2020) while the opposite pattern was observed for South African and European locations (Maier et al., 2018; Glaser et al., 2022). Glaser et al. (2022) speculated that this contrast in microbial richness patterns may be linked to differences in the way moisture is supplied in these ecosystems (rain versus snow versus fog). This suggests that microbial diversity depends on local and clade specific drivers and stressors, highlighting the role in which landscape patterns condition the composition and structure of soil surface microbes. Such compositional differences may result in different rates of microbial mediated biogeochemical processes and topsoil aggregate stability. However, there are too few studies yet to draw general conclusions.

More than ¾ of all Cyanobacteria ASVs belonged to the order Oscillatoriales with high representation in the genera *Microcoleus*, *Allocoleopsis*, *Crinalium*, *Pycnacronema*, and *Parifilum*. An additional 11% of the relative abundance in Cyanobacteria were filamentous Synechococcales with *Phormidesmis* in the family Leptolyngbyaceae, *Trichocoleus* in Trichocoleusaceae and oculatellacean taxa being abundant (Supplementary Table 5). These taxa are known to have bundle forming abilities and/or to produce high amounts of exopolysaccharides (Garcia-Pichel and Wojciechowski 2009; Mühlsteinová et al., 2014a; Osorio-Santos et al., 2014; Martins et al., 2018; Mikhailyuk et al., 2019; Fernandes et al., 2021), both of which improve soil surface aggregation and consequently soil stability in the vegetation-poor plant interspaces in drylands (Garcia-Pichel and Wojciechowski 2009; Büdel et al., 2016). Our study adds to an extant body of literature that highlights the dominance of exopolysaccharide forming cyanobacteria in desert topsoil (see review by Büdel et al., 2016). A particularly interesting result within Oscillatoriales was the differences in Microcoleaceae and Coleofasciculaceae taxa abundances on landforms (Figure 3). Past studies have shown temperature preferences of these two families with *Microcoleus vaginatus* (Microcoleaceae) being psychrotolerant and more abundant in cold deserts while members of the Coleaofasciculaceae were thermotolerant and more abundant in hot deserts (Garcia-Pichel et al., 2013). However, geomorphic difference in abundance within the same climatic regime is a novel finding warranting further investigations about the possible mechanism behind this habitat selection.

Cyanobacteria are also the microbial group with the most detailed and complete taxonomic classification of all cyanobacterial ASVs identified, with many taxa assigned to recently described or revised cyanobacteria (Figure 2; Supplementary Figure 1). Immense progress has been made in the past 3 decades examining and describing soil cyanobacteria diversity in drylands using the polyphasic approach (see recent work by Mühlsteinová et al., 2014a,b; Pietrasiak et al., 2014a,b, 2019, 2021; Osorio-Santos et al., 2014; Bohunická et al., 2015; Becerra-Absalón et al., 2019; Jung et al., 2020b; Fernandes et al., 2021; Baldarelli et al., 2022). This underlines the importance of taxonomic studies in microbiology in the current era, as newly erected or revised microbial species are linked to DNA based phylogenetic benchmarks, which in turn allows for improved operational taxonomic units (OTU) and ASV classification obtained in next generation sequencing surveys. However, this study still demonstrates the potential of discovering unclassifiable clades of cyanobacteria up to the order level, and thus underscores how much more taxonomic work remains to be done.

Archaea was least diverse in phylogenetic and species diversity dominated by only three phyla and six families (Supplementary Table 6). However, we detected the presence of taxa important for carbon and nitrogen cycling. Nitrososphaeraceae, phylum Thaumarcheota, had an extremely high abundance in all soils with over 98% relative abundance, except for P-COLL where abundance dropped to 95%. Our ASVs related closely with *Nitrososphaera* and *Nitrosocosmicus* spp., both ammonia-oxidizing archaea. These two genera are biogeochemically interesting as they contribute to nitrification in soils via ammonia oxidation pathways, but also in the process fix carbon dioxide via chemolithotrophy (Lehtovirta-Morley et al., 2016; Alves et al., 2019; Sauder et al., 2020). However, their role in dryland carbon cycling has not been explored yet. In P-COLL *Methanobacterium*, phylum Euryarcheota, and a few unclassifiable haloarchaeote and euryarchaeote ASVs were found with >1% relative abundance, indicating ephemerally ponding and subsequent anoxic conditions.

The fungal community of Jornada surface soils was dominated by Dothideomycetes (Ascomycota) and Agaricomycetes (Basidiomycota), supporting findings by Porras-Alfaro et al. (2011) from central New Mexico biocrusts as well as by Mueller et al. (2015) and Pombubpa et al. (2020) from Mojave Desert biocrusts. We found an average of 46.3% of all fungal ASV belonged to an unknown family in class Dothideomycetes, despite being one of the most studied fungal families (Schoch et al., 2009; McKenzie et al., 2014). In addition, 21% of fungal ASVs were completely unknown; that is, we do not know their taxonomic identification at the class level (Supplementary Table 7). Interestingly, the Agaricomycetes, which house some of the few desert-adapted mushrooms, such as *Montagnea* spp., were consistently found in all sites. The Eurotiomycetes were found in high abundance in three of the sites that were visually most covered with biocrust and also had the highest lichen biocrust cover (P-TOBO, T-TAYL, and T-WEST, Supplementary Table 6). Eurotiomycetes contain many lichen fungi (family Verrucariaceae) which are an essential component of biocrusts. Lecanoromycetes lichen fungi were most abundant at T-TAYL. Black yeast fungi in the Eurotiomycetes also include Chaetothyriales, which are common in lichen dominated biocrusts as well (Warren et al., 2019; Pombubpa et al., 2020; Carr et al., 2021). Sordariomycetes were present in all sites, reaching their highest abundances in the playa sites (P-COLL and P-SMAL). Many Sordariomycetes fungi are melanized (e.g., dark septate endophytic fungi and lichenicolous fungi) which allows them to withstand stressful conditions, such as high radiation (Grishkan, 2011; Muggia and Grube, 2018; Wong et al., 2019), likely prevalent in playa sites which lack perennial vegetation. Similarly, Mortierellomycetes relative abundances were highest in playa samples. Mortierellomycetes houses species known to be endophytic (Wani et al., 2017) and to produce lipid compounds, which can confer stress tolerance (Lu et al., 2020). Finally, the Tremellomycetes (phylum Basidiomycota) were found consistently in shrub dominated sites (Creosote, Mesquite, and Tarbush) while Pezizomycetes (phylum Ascomycota) were slightly more abundant in grass dominated sites compared to shrub dominated sites (Supplementary Table 3), which could indicate class-level co-occurrences in the root zones of these two fundamentally different plant functional groups (Brunel et al., 2020).

### 4.2. Landscape features shape soil surface microbiomes

Our prediction that vegetation type would structure the soil surface microbiomes predominantly over soil and landscape features was not strongly supported. In contrast, in our study landscape features such as landforms, ecological sites and states and associated soil properties were either equally or more important in explaining the variability in the soil microbiome data (Table 2; Supplementary Table 1). The soil geomorphic template has been shown to be predictive for vegetation and animal community composition and structure (Monger and Bestelmeyer, 2006; Peters et al., 2006; Monger et al., 2015). Our study demonstrated for the first time in the Chihuahuan Desert how dryland landscapes can also harbor unique assemblages of soil surface microbes. For example, playas had distinct soil surface microbiomes, which differed from alluvial flat soil microbiomes, which in turn were distinct from alluvial plain and fan piedmont soil microbiomes. Like other basin and range landscapes in the United States, the Jornada Basin and Range is divided into mountains and hills (not included in this study), the piedmont slope, and the basin floor. The three landforms: playa, alluvial flat, and alluvial plain were part of the basin floor, while one landscape class, fan piedmont, represented part of the piedmont slope. Thus, playas and alluvial flats occur on topographically low areas of hydrologically closed catchments. Precipitation and run-on from upland areas during large rainfall events transport water and nutrients through fan piedmonts and alluvial plains, onto alluvial flats and playas via sheet flooding—which in turn collects water, nutrients, and fine texture particles on both these landforms (Mckenna and Sala, 2018). Although topographically similar, playas are typically more barren, finer textured, and will ephemerally pond while the alluvial flat landforms typically do not pond, are loamier, and are shrub dominated. The fan piedmonts occur at higher topographic positions than the basin floor, have coarser textured soils, and are exposed to different hydrological and erosional forces compared to basin landforms. These distinct abiotic conditions across landforms suggest the presence of different niches for soil microbes, which thus links to characteristic microbiomes.

Our findings demonstrate how investigating microbiome data within the landscape context could elucidate how geomorphic processes such as landscape evolution, connectivity, and water, wind, animal, and disturbance dispersal may link to soil and biocrust microbiome composition, structure, and ultimately functioning at a much deeper level and across different scales. Geomorphology effects have been documented to influence microbial meta-communities at a patch scale predicting biocrust functional group mosaics (Pietrasiak et al., 2013, 2014a,b; Williams et al., 2013) or identifying differences in biogeochemical cycling in shrub island—plant interspace patterns (Schlesinger et al., 1996) with consequences to ecosystem multifunctionality. However, disentangling spatial drivers of soil microbiome assembly remains challenging, and so does analysis of their feedback to ecosystem functions in drylands. As Landform was the most important predictor for Bacteria, Cyanobacteria and Archaea richness, relative abundances, species turnover, and community composition in our study, future studies should explore how these patterns relate to the various ecosystem services microbes provide in dryland ecosystems.

### 4.3. A suite of soil properties linked to landscapes structure relate to surface soil microbiomes

No single soil physical or chemical property was found to be significant for predicting microbial diversity, community composition, and structure. Rather it was the combination of variables that were important to surface soil microbiomes at Jornada Basin. Results showed that gravel content, texture, moisture, pH, and salt content (estimated via electrical conductivity) all played an important role in shaping the soil surface microbiome. Previous studies have indicated that fine textured soils promote surface soil microbial communities compared to those with high sand content (Read et al., 2008; Pietrasiak et al., 2011a; Stovall et al., 2022). This relationship may exist because the small silt and clay particles of fine textured soils provide a more beneficial habitat for soil organisms, with their higher nutrient and water holding capacities as well as pH buffering and cation exchange properties. A novel finding of our study was that the observed interplay of measured soil parameters and surface soil microbiome characteristics was linked to the soil geomorphic template of Monger and Bestelmeyer (2006). Finer textured soils were associated with playa and alluvial flat sites. These soils recorded higher soil moisture, presumably held in the finer soil pores from the preceding rain event for much longer compared to coarse textured soils. Also, these finer textured soils recorded higher electrical conductivity and pH values. This suite of soil properties harbored unique surface soil microbial communities distinctly different from the coarser textured soils of alluvial plain and fan piedmont sites.

Although soil parent material was an important predictor for community composition, our study lacked representative sampling across the diverse Jornada Basin and Range parent materials and was weakened by a small sample size for some substrates, limiting detailed geologic interpretation for the heterogeneous Jornada basin and piedmont landscape. Most of the NPP sites formed in alluvium parent material derived from felsic and intermediate igneous rocks of the Doña Ana mountains or were derived from ancestral river deposits of the Rio Grande (Michaud et al., 2013). Identified PERMANOVA differences (Table 2) and NMDS ordination clustering (Figure 4) in the cyanobacterial and fungal microbiome compositions appear linked to the difference in alluvium’s mixed mineralogy. Previous studies in the North American drylands have demonstrated how mineralogy impacts biocrust abundance and composition with gypsum and monzo-granitic parent material promoting biocrust diversity and coverage (Bowker and Belnap, 2008; Pietrasiak et al., 2011a; Bowker et al., 2016). However, more studies are needed to investigate these impacts on the dryland soil microbiome. Future studies may need to employ a stratified methodology, to examine in more detail soil mineralogical influences to dryland microbial communities.

### 4.4. Disturbance

Many previous studies have found that physical disturbances to desert areas can have detrimental impacts on the soil microbial communities and the broader desert environment resulting in loss of diversity and diminished ecosystem functions (Belnap and Gillette, 1998; Harper and Belnap, 2001; Thomas and Dougill, 2006; Pietrasiak et al., 2011b; Concostrina-Zubiri et al., 2014; Pointing and Belnap, 2014; Ferrenberg et al., 2015). Particularly, soil surface microbiomes are most vulnerable to many forms of disturbance, as they establish on the topmost layer of soil (Nelson et al., 2022). In our study, the trampling and compacting disturbance caused by researchers monitoring aboveground NPP and soil moisture was either not severe enough to cause significant observable changes in any DNA based microbial and soil characteristics we investigated, e.g., indicating resistance, or reflect a degree of resilience at the applied level of disturbance.

A large body of studies exist investigating the impact of high frequency or high impact trampling disturbance treatments to biocrust abundance and functional group diversity in drylands (see reviews of Pointing and Belnap, 2014; Zaady et al., 2014; Li et al., 2021) while we still know little about microbiome responses to disturbance gradients and what the disturbance thresholds are that cause state changes in microbiomes. Resilience of microbial communities can be supported for instance by nearby undisturbed soil serving as a propagule source which can promote rapid recolonization after a trampling event. In the case of motile prokaryotes, such as *Microcoleus*, *Trichocoleus*, and certain other filamentous Cyanobacteria, it could also result from their ability to periodically find shelter a few millimeters to centimeters below the surface. In other locations, surrounding areas with healthy biocrust communities have been postulated to contribute to the spread of biocrusts organisms into areas that have been previously disturbed (Bowker, 2007). Also, DNA based microbiome studies may not be sensitive enough to detect short-term microbial responses to disturbance, but RNA based studies may reveal more rapid responses based on changes to microbial gene expression activity. Future studies could implement disturbance gradients and conduct manipulative experiments, applying meta-transcriptomics and metabolomics to quantify and tease apart microbiome resistance versus resilience. Such investigations will aid to understand how trampling, off-road vehicle, and grazing disturbance affects the microbial communities more clearly in the Chihuahuan Desert and how and when state transitions occur in dryland soil microbiomes.

### 4.5. Implications for dryland ecology

Baseline knowledge of surface soil microbial communities and subsequent long-term monitoring of their composition and structure in diverse Chihuahuan Desert soil types and landforms could inform resource management and restoration efforts in novel ways. Surface soil microbial communities are instrumental to numerous ecological processes in drylands including soil stability, carbon sequestration, bioweathering and nutrient cycling, and water dynamics (Harper and Belnap, 2001; Belnap et al., 2013; Pietrasiak et al., 2013). They also can act as first responder to stressors right at the interface of the atmosphere, hydrosphere, geosphere, and biosphere. Despite their critical roles, bottom-up impacts, and existence at a critical zone in dryland ecosystems, we have only recently begun to integrate soil microbiomes as elements in dryland management and restoration (Velasco Ayuso et al., 2016; Maestre et al., 2017; Bethany et al., 2021) and many knowledge gaps remain. For example, we often lack comprehensive compositional knowledge of soil microbiomes from potential reference sites, needed to compare undisturbed surfaces to altered and degraded sites within expansive areas of the Southwestern United States. Furthermore, it is unclear what microbial population boundaries might apply at many locations. Characterizing the native soil microbiomes in a systematic way, informed by the landscape’s heterogeneous properties, will improve our understanding of basic community and population ecological patterns. This in turn will result in more informed inoculation efforts as viable rehabilitation tactics for different disturbed areas with potentially distinct microbiomes and variable mixtures of abiotic factors (Bowker, 2007).

Further, understanding what factors influence the dynamics of these communities could also prove vital in understanding how to preserve our dryland soils and hence our natural desert areas (Bowker, 2007; Antoninka et al., 2020), potentially saving billions of dollars that are currently lost annually due to desertification and unintended vegetation state transitions (Bowker et al., 2005). Yet, soil microbiome and biocrust restoration success is still challenged by many factors including loss of inoculum due to erosional processes and inoculum short- and long-term survival (Antoninka et al., 2020; Bowker et al., 2020; Zhou et al., 2020). Some of these challenges may need to be tackled with an improved soil geomorphic knowledge base, which could then inspire and inform novel strategies of ecological restoration. For example, a yet unrecognized cause of biocrust restoration failures, evidenced by non-viable microbial populations after inoculation at existing sites, could be a link to geomorphology. Biocrust microbial inocula derived from differing geomorphic origins may introduce microbial populations not adapted to local landscape settings and thus lead to inoculum establishment failure. We therefore recommend the selection process of source locations for microbial inocula to include consideration of the concept of the soil geomorphic template, with source locations corresponding to a similar landform setting, ecological site, and soil mineralogy.

## 5. Conclusion

The composition and distribution patterns of soil microbial communities in biocrust are not well understood in desert ecosystems, nor are those of other free-living soil microbial communities living at the soil surface. Our study provides one of the most comprehensive soil microbial surveys performed to date in the Chihuahuan Desert, investigating microbes of all three domains in the tree of life. We revealed unique, diverse and poorly characterized surface soil microbiomes. Many of the detected microbes are first records for the region. Land surface properties, soil characteristics, and the influence of trampling disturbance explained certain aspects of the observed variation in soil microbial community composition and structure. We also discovered that overall landscape features over vegetation and soil properties were most important predictors of surface soil microbial community composition and structure in this dryland landscape. Alpha diversity did not change as much as relative abundances of specific organismal groups, community composition, and species turnover. We observed that relative abundance changes were distinct at phyla level but also at finer taxonomic resolution of individual microbial phyla. Much may get overlooked if studies focus only at the phyla level without exploring responses in detail within key microbial groups, such as in our case Cyanobacteria, Thaumarcheota, and Fungi. Gravel content, soil moisture, texture, pH, EC, and lichen biocrust cover associated strongly with the environmental gradients structuring the soil surface communities. This information could be used to initiate experimental studies in which soil properties are directly manipulated and the resulting soil microbial community responses are observed. Understanding these drivers would then provide a basis from which to approach soil microbial restoration and recovery in degraded desert areas. Additionally, our characterization of the soil microbial communities from a protected and preserved area of the Chihuahuan Desert can be used by future studies as a first reference framework for what healthy and natural soil communities consist of in the Chihuahuan Desert.

## Data availability statement

The datasets presented in this study can be found in online repositories. The MiSeq generated raw sequence reads of our amplified 16S and ITS rRNA gene markers were submitted to the NCBI GenBank Sequence Read Archive database associated with BioProject accession number PRJNA867662. Analysis scripts for UNIX tool processing of raw sequence data can be found at GitHub (https://github.com/stajichlab/Microbiome_Jornada/). Metadata including soils data for this study can be found in the metadata (MIMS) associated with our BioProject PRJNA867662, in the Supplemental Material, and referenced data packages. R scripts can be accessed at https://github.com/stajichlab/Microbiome_Jornada.

## Author contributions

FH, JA, and NiP designed the study. FH collected and processed the soil samples for chemical and physical analyses as well as performed DNA extraction under guidance of NiP. FH and NuP ran barcode PCR and prepared the libraries for 16S rRNA and ITS1 amplicons. JS provided bioinformatics logistics support. JS and NuP established the bioinformatics pipelines, and NuP, AD, and FH subsequently processed sequence data. AD curated the data and deposited them in NCBI. CM and DJ performed statistical analyses with input from NiP. NiP (cyanobacteria), AR-O (fungi), JS (fungi), and SS (soil and geomorphology) provided expertise in data interpretation. FH’s undergraduate honor thesis served as the first draft of the manuscript. All authors contributed to the article and approved the submitted version.

## Funding

FH was supported by Howard Hughes Medical Institute’s 2014 Science Education grant 52008103 to New Mexico State University and the Research Experience for Undergraduate Student Summer Fellowship awarded through the Jornada Long Term Ecological Research Station Research program (NSF DEB 1832194). The Ecological Society of America and the Native Plant Society of New Mexico provided additional research funding that made this study possible. NuP was awarded the Jornada Basin LTER Graduate Student Summer Fellowship (NSF DEB 1832194), which led to the development of the next generation amplicon sequencing bioinformatics pipeline.

## Conflict of interest

The authors declare that the research was conducted in the absence of any commercial or financial relationships that could be construed as a potential conflict of interest.

## Publisher’s note

All claims expressed in this article are solely those of the authors and do not necessarily represent those of their affiliated organizations, or those of the publisher, the editors and the reviewers. Any product that may be evaluated in this article, or claim that may be made by its manufacturer, is not guaranteed or endorsed by the publisher.
