# Supplementary material for: Landscape characteristics shape surface soil microbiomes in the Chihuahuan Desert
Source: Front Microbiol. 2023 Jun 7;14:1135800. doi: 10.3389/fmicb.2023.1135800 (PMC10282155; doi:10.3389/fmicb.2023.1135800)

**SUPPLEMENTAL MATERIAL**

Supplement Table 1: Classifications of study sites. Green column headers denote classification used in the analysis.

| **Site** | **Vegetation Zone** | **Landscape (Monger)** | **Landscape (Hansen et al.)** | **Landform (Monger)** | **Landform**  **(Hansen et al.)** | **Parent Material (Monger)** | **Parent Material** | **Ecological Site** | **Ecological State** |  |
| --- | --- | --- | --- | --- | --- | --- | --- | --- | --- | --- |
| C-CALI | Creosote | Piedmont Slope (100%) | Piedmont Slope | Fan Piedmont (100%) | Fan Piedmont | Alluvium from Igneous Bedrock | Igneous alluvium | Gravelly | shrubland with ERLE |  |
| C-GRAV | Creosote | Piedmont Slope (100%) | Piedmont Slope | Fan Piedmont (100%) | Fan Piedmont | Alluvium from Igneous Bedrock | Igneous alluvium | Gravelly | shrubland |  |
| C-SAND | Creosote | Piedmont Slope (100%) | Piedmont Slope | Fan Piedmont (100%) | Fan Piedmont | Alluvium from Igneous Bedrock | Igneous alluvium | Gravelly sand | shrubland with ERLE |  |
| G-BASN | Grassland | Piedmont Slope (100%) | Piedmont Slope | Fan Piedmont (100%) | Fan Piedmont | Alluvium from Igneous Bedrock | Igneous alluvium | Loamy | reference grassland |  |
| G-IBPE | Grassland | Basin Floor (100%) | Basin Floor | Alluvial Plain Wind Worked (100%) | Alluvial Plain | Sediments Derived from the Ancestral Rio Grande | Ancestral Rio Grande alluvium | Sandy | shrub- invaded |  |
| G-SUMM | Grassland | Piedmont Slope (92%) | Piedmont Slope | Alluvial Fan Collar and Pediment (92%) | Fan Piedmont | Alluvium from Igneous Bedrock | Igneous alluvium | Gravelly sand | exotic- invaded |  |
|  |  |  |  |  |  |  |  |  |  |  |
|  |  | Mountain & Hills (8%) |  | Bedrock Outcrop (8%) |  |  |  |  |  |  |
|  |  |  |  |  |  |  |  |  |  |  |
| M-NORT | Mesquite | Basin Floor (100%) | Basin Floor | Alluvial Plain Reddish Brown Sand Sheet (97%) | Alluvial Plain | Sediments Derived from the Ancestral Rio Grande | Ancestral Rio Grande alluvium | Sandy | shrubland |  |
|  |  |  |  | Alluvial Plain Wind Worked (3%) |  |  |  |  |  |  |
| M-RABB | Mesquite | Basin Floor (100%) | Basin Floor | Alluvial Plain Wind Worked (100%) | Alluvial Plain | Sediments Derived from the Ancestral Rio Grande | Ancestral Rio Grande alluvium | Sandy | shrubland |  |
| M-WELL | Mesquite | Basin Floor (100%) | Basin Floor | Alluvial Plain Wind Worked (100%) | Alluvial Plain | Sediments Derived from the Ancestral Rio Grande | Ancestral Rio Grande alluvium | Sandy | shrubland |  |
| P-COLL | Playa | Basin Floor (100%) | Basin Floor | Playa (100%) | Playa | Sediments Derived from the Ancestral Rio Grande | Rio Grande playa alluvium | Playa/ Bottomland | altered grassland |  |
| P-SMAL | Playa | Basin Floor (100%) | Basin Floor | Playa (100%) | Playa | Sediments Derived from the Ancestral Rio Grande | Rio Grande playa alluvium | Playa/ Bottomland | altered grassland |  |
| P-TOBO | Playa | Basin Floor (100%) | Basin Floor | Lake-Plain Playa (100%) | Alluvial Flat | Gypsiferous Sediments | Gypsiferous Sediments | Bottomland | reference grassland |  |
| T-EAST | Tarbush | Basin Floor (100%) | Basin Floor | Alluvial Flat (100%) | Alluvial Flat | Alluvium from Igneous Bedrock | Igneous alluvium | Loamy | shrub- invaded |  |
| T-TAYL | Tarbush | Piedmont Slope (100%) | Piedmont Slope | Fan Piedmont (100%) | Fan Piedmont | Alluvium from Sedimentary Bedrock | Sedimentary alluvium | Loamy | shrub- invaded |  |
| T-WEST | Tarbush | Basin Floor (100%) | Basin Floor | Alluvial Flat (100%) | Alluvial Flat | Alluvium from Igneous Bedrock | Igneous alluvium | Loamy | shrub- invaded |  |

Supplement Table 2: Climate, aboveground plant biomass, and lichen cover data associated with the 15 NPP study sites.

| **Site** | **Vegetation zone** | **Soil Sampling Date** | **Maximum Daily Air Temperature (°C)** | **Amount of Most Recent Precipitation Event (mm)** | **Days Prior to Sampling on which Precipitation occurred** | **Soil Volumetric Water Content at 10cm depth (%)** | **Spring 2016 Estimated Perennial Grass Biomass (g*m^-2^)** | **Spring 2016 Estimated Woody Shrub Biomass (g*m^-2^)^*^** | **Spring 2016 Lichen Crust Cover (%)** |
| --- | --- | --- | --- | --- | --- | --- | --- | --- | --- |
| C-CALI | Creosote | June 22, 2016 | 40.90 | 5.00 | 4 | 0.00% | 1.50 | 75.44 | 0.00 |
| C-GRAV | Creosote | June 22, 2016 | 40.90 | 3.90 | 37 | 1.25% | 3.34 | 120.54 | 0.00 |
| C-SAND | Creosote | June 24, 2016 | 38.21 | 1.20 | 14 | 0.85% | 3.84 | 104.51 | 0.00 |
| G- BASN | Grassland | June 23, 2016 | 40.02 | 0.40 | 13 | 5.30% | 49.10 | 7.10 | 0.00 |
| G-IBPE | Grassland | July 5, 2016 | 40.44 | 3.70 | 4 | 0.00% | 44.53 | 23.08 | 0.00 |
| G-SUMM | Grassland | July 5, 2016 | 38.73 | 5.40 | 4 | 4.60% | 13.72 | 5.53 | 0.00 |
| M-NORT | Mesquite | July 6, 2016 | 39.24 | 4.00 | 5 | 2.70% | 0.80 | 80.89 | 0.00 |
| M-RABB | Mesquite | July 6, 2016 | 29.69 | 5.20 | 5 | 2.60% | 10.70 | 57.23 | 0.00 |
| M-WELL | Mesquite | July 6, 2016 | 38.54 | 0.90 | 5 | 1.00% | 0.78 | 112.74 | 0.00 |
| P-COLL | Playa | July 5, 2016 | 40.62 | 4.80 | 4 | 8.95% | 1.12 | 0.00 | 0.00 |
| P-SMAL | Playa | June 23, 2016 | *N/A* | *N/A* | *N/A* | *N/A* | 1.72 | 0.00 | 0.00 |
| P-TOBO | Playa | June 22, 2016 | 40.98 | 0.50 | 21 | 18.15% | 0.25 | 0.00 | 0.61 |
| T-EAST | Tarbush | June 16, 2016 | 36.42 | 0.20 | 11 | 8.80% | 17.06 | 53.86 | 0.61 |
| T-TAYL | Tarbush | June 22, 2016 | 40.98 | 0.70 | 15 | 5.90% | 2.23 | 18.28 | 16.94 |
| T-WEST | Tarbush | June 16, 2016 | 36.74 | 14.60 | 15 | 20.50% | 8.60 | 15.22 | 1.02 |

*Does not include cacti or *Yucca* species.

Supplement Table 3: Variable importance ranked by AICc results.

| **Group** | **Model** | **K** | **AICc** | **Delta AICc** | **AICcWt** | **notes** |
| --- | --- | --- | --- | --- | --- | --- |
| **Total Bacteria** | Landscape | 3 | 223.868 | 0.000 | 0.290 | Landscape comprises 29% of the AICc Weight but is with 2 AICc units of the Null model |
|  | Landform | 5 | 223.955 | 0.087 | 0.278 | Landform comprises 27.8% of the AICc Weight but is with 2 AICc units of the Null model |
|  | Vegetation Zone | 6 | 224.072 | 0.204 | 0.262 | Vegetation Zone comprises 26.2% of the AICc Weight but is with 2 AICc units of the Null model |
|  | **null** | 2 | 224.936 | 1.068 | 0.170 | Null model comprises 17.00% of the AICc Weight |
|  | Parent Material | 6 | 235.899 | 12.030 | 0.001 | worse than Null model |
|  | Ecological Site | 7 | 242.592 | 18.723 | 0.000 | worse than Null model |
|  | Ecological State | 7 | 245.632 | 21.764 | 0.000 | worse than Null model |
| **Cyano-**  **bacteria** | **Landform** | 5 | 119.669 | 0.000 | 1.000 | Landform comprises 100% of the AICc Weight |
|  | Vegetation Zone | 6 | 142.148 | 22.480 | 0.000 | Within 2 AICc units of Null model |
|  | **null** | 2 | 142.683 | 23.014 | 0.000 | Within 2 AICc units of Null model |
|  | Landscape | 3 | 144.133 | 24.464 | 0.000 | Within 2 AICc units of Null model |
|  | Ecological Site | 7 | 147.014 | 27.346 | 0.000 | worse than Null model |
|  | Ecological State | 7 | 147.093 | 27.424 | 0.000 | worse than Null model |
|  | Parent Material | 6 | 153.141 | 33.473 | 0.000 | worse than Null model |
| **Archaea** | **Landform** | 5 | 96.124 | 0.000 | 0.985 | Landform comprises 98.51% of the AICc Weight |
|  | Vegetation Zone | 6 | 105.577 | 9.453 | 0.009 | Vegetation is within 2 AICc units of the Null Model |
|  | null | 2 | 106.870 | 10.746 | 0.005 |  |
|  | Landscape | 3 | 109.417 | 13.293 | 0.001 | worse than Null model |
|  | Ecological Site | 7 | 112.118 | 15.994 | 0.000 | worse than Null model |
|  | Parent Material | 6 | 117.974 | 21.850 | 0.000 | worse than Null model |
|  | Ecological State | 7 | 124.585 | 28.461 | 0.000 | worse than Null model |
| **Fungi** | **null** | 2 | 186.678 | 0.000 | 0.615 | no model performed better than Null; Null has 61.5% of the AICc Weight |
|  | Landscape | 3 | 187.694 | 1.015 | 0.370 | worse than Null model |
|  | Landform | 5 | 194.203 | 7.524 | 0.014 | worse than Null model |
|  | Vegetation Zone | 6 | 200.204 | 13.526 | 0.001 | worse than Null model |
|  | Parent Material | 6 | 201.795 | 15.116 | 0.000 | worse than Null model |
|  | Ecological Site | 7 | 201.908 | 15.230 | 0.000 | worse than Null model |
|  | Ecological State | 7 | 208.643 | 21.964 | 0.000 | worse than Null model |

Supplement Table 4: Relative abundance by Bacteria phyla.

| Taxo-  nomic  rank | Taxo-  nomic  name | P-COLL | P-SMAL | P-TOBO | T-EAST | T-WEST | T-  TAYL | M-WELL | M-RABB | M-NORT | G-BASN | G-IBPE | G-SUMM | C-SAND | C-CALI | C-GRAV | **Average** |
| --- | --- | --- | --- | --- | --- | --- | --- | --- | --- | --- | --- | --- | --- | --- | --- | --- | --- |
| Phylum | Proteo-bacteria | 32% | 43% | 21% | 23% | 21% | 23% | 29% | 33% | 34% | 29% | 29% | 37% | 31% | 28% | 26% | **29%** |
| Phylum | Actino-bacteria | 19% | 25% | 35% | 32% | 37% | 24% | 34% | 24% | 29% | 24% | 24% | 19% | 37% | 39% | 29% | **29%** |
| Phylum | Chloro-flexi | 9% | 11% | 12% | 12% | 16% | 18% | 9% | 8% | 7% | 12% | 10% | 6% | 8% | 11% | 13% | **11%** |
| Phylum | Acido-bacteria | 13% | 8% | 5% | 6% | 5% | 8% | 7% | 12% | 8% | 11% | 10% | 12% | 7% | 6% | 9% | **8%** |
| Phylum | Bactero-idetes | 11% | 3% | 3% | 4% | 2% | 3% | 6% | 10% | 7% | 6% | 10% | 11% | 4% | 4% | 6% | **6%** |
| Phylum | Gemma-timonadetes | 5% | 3% | 5% | 4% | 4% | 4% | 4% | 4% | 5% | 5% | 5% | 4% | 4% | 5% | 4% | **4%** |
| Phylum | Plancto-mycetes | 5% | 3% | 5% | 5% | 6% | 8% | 3% | 3% | 3% | 5% | 3% | 4% | 3% | 3% | 4% | **4%** |
| Phylum | Cyano-bacteria | 1% | 0% | 10% | 10% | 5% | 5% | 3% | 1% | 1% | 1% | 2% | 0% | 2% | 0% | 1% | **3%** |
| Phylum | Verruco  microbia | 3% | 1% | 0% | 1% | 0% | 2% | 1% | 1% | 1% | 2% | 2% | 2% | 1% | 1% | 2% | **1%** |
| Phylum | Armatimo-nadetes | 1% | 0% | 1% | 1% | 1% | 1% | 1% | 1% | 1% | 2% | 1% | 1% | 1% | 1% | 2% | **1%** |
| Phylum | <1% abund. | 2% | 2% | 3% | 2% | 3% | 2% | 4% | 2% | 3% | 3% | 5% | 3% | 3% | 3% | 4% | **3%** |

Supplement Table 5: Relative abundance within Phylum Cyanobacteria.

| Taxo-  nomic  rank | Taxo-  nomic  name | P-COLL | P-SMAL | P-TOBO | T-EAST | T-WEST | T-  TAYL | M-WELL | M-RABB | M-NORT | G-BASN | G-IBPE | G-SUMM | C-SAND | C-CALI | C-GRAV | **Average** |
| --- | --- | --- | --- | --- | --- | --- | --- | --- | --- | --- | --- | --- | --- | --- | --- | --- | --- |
| Order | Oscillatoria-les | 38% | 53% | 90% | 88% | 84% | 62% | 90% | 95% | 74% | 93% | 80% | 82% | 97% | 71% | 76% | **78%** |
| Order | Synechococ-cales | 29% | 30% | 4% | 4% | 6% | 31% | 5% | 1% | 8% | 2% | 12% | 11% | 1% | 6% | 14% | **11%** |
| Order | Unclassif. order | 1% | 13% | 3% | 5% | 7% | 3% | 4% | 3% | 12% | 3% | 7% | 4% | 1% | 6% | 10% | **5%** |
| Order | Nostocales | 32% | 1% | 3% | 2% | 4% | 4% | 0% | 1% | 2% | 1% | 0% | 0% | 0% | 0% | 0% | **3%** |
| Order | Chroococci-diopsidales | 0% | 3% | 0% | 0% | 0% | 1% | 1% | 0% | 2% | 1% | 1% | 4% | 1% | 12% | 1% | **2%** |
| Order | <1% abund. | 0% | 0% | 0% | 0% | 0% | 0% | 0% | 0% | 2% | 0% | 0% | 0% | 0% | 6% | 0% | **1%** |
| Family | Microcolea-ceae | 11% | 14% | 52% | 32% | 37% | 21% | 72% | 84% | 42% | 50% | 20% | 39% | 94% | 47% | 68% | **46%** |
| Family | Coleofasci-culaceae | 7% | 25% | 37% | 55% | 46% | 40% | 13% | 4% | 1% | 41% | 58% | 25% | 3% | 0% | 6% | **24%** |
| Family | Leptolyng-byaceae | 18% | 22% | 0% | 1% | 1% | 28% | 2% | 1% | 4% | 1% | 7% | 11% | 1% | 6% | 5% | **7%** |
| Family | unclassified family | 1% | 13% | 3% | 5% | 7% | 3% | 4% | 3% | 12% | 3% | 7% | 4% | 1% | 6% | 10% | **5%** |
| Family | Gomontiel-laceae | 0% | 0% | 0% | 0% | 0% | 0% | 3% | 7% | 31% | 1% | 0% | 7% | 0% | 24% | 0% | **5%** |
| Family | Oscillatoria-ceae | 19% | 3% | 1% | 2% | 1% | 0% | 2% | 0% | 0% | 1% | 1% | 7% | 0% | 0% | 1% | **2%** |
| Family | Trichocole-usaceae | 0% | 2% | 1% | 1% | 0% | 1% | 3% | 0% | 4% | 1% | 4% | 0% | 0% | 0% | 9% | **2%** |
| Family | Chroococci-diopsidaceae | 0% | 3% | 0% | 0% | 0% | 1% | 1% | 0% | 2% | 1% | 1% | 4% | 1% | 12% | 1% | **2%** |
| Family | Scytonema-taceae | 8% | 1% | 3% | 2% | 4% | 3% | 0% | 1% | 2% | 1% | 0% | 0% | 0% | 0% | 0% | **2%** |
| Family | Nostocaceae | 18% | 0% | 0% | 0% | 0% | 0% | 0% | 0% | 0% | 0% | 0% | 0% | 0% | 0% | 0% | **1%** |
| Family | Oculatella-ceae | 9% | 6% | 0% | 0% | 0% | 0% | 0% | 0% | 0% | 0% | 0% | 0% | 0% | 0% | 0% | **1%** |
| Family | unclassified family | 0% | 11% | 0% | 0% | 0% | 0% | 0% | 0% | 0% | 0% | 0% | 4% | 0% | 0% | 1% | **1%** |
| Family | <1% abund. | 8% | 0% | 2% | 2% | 4% | 3% | 0% | 0% | 2% | 0% | 0% | 0% | 0% | 6% | 0% | **2%** |
| Genus | *Microcoleus* | 1% | 12% | 50% | 31% | 34% | 19% | 72% | 83% | 42% | 50% | 20% | 39% | 93% | 47% | 68% | **44%** |
| Genus | *Allocoleopsis* | 0% | 23% | 13% | 5% | 13% | 5% | 8% | 1% | 0% | 21% | 33% | 0% | 0% | 0% | 0% | **8%** |
| Genus | unclassified genus | 7% | 2% | 10% | 27% | 9% | 15% | 2% | 1% | 0% | 2% | 11% | 0% | 0% | 0% | 0% | **6%** |
| Genus | *Crinalium* | 0% | 0% | 0% | 0% | 0% | 0% | 3% | 7% | 31% | 0% | 0% | 4% | 0% | 24% | 0% | **5%** |
| Genus | *Pycnacro-nema* | 0% | 0% | 2% | 5% | 8% | 3% | 3% | 2% | 0% | 3% | 12% | 21% | 2% | 0% | 6% | **5%** |
| Genus | unclassified genus | 1% | 13% | 3% | 5% | 7% | 3% | 1% | 0% | 1% | 3% | 7% | 0% | 0% | 6% | 9% | **4%** |
| Genus | unclassified genus | 9% | 19% | 0% | 0% | 1% | 12% | 2% | 1% | 3% | 0% | 5% | 0% | 0% | 0% | 3% | **4%** |
| Genus | *Parifilum* | 0% | 0% | 6% | 7% | 7% | 6% | 0% | 0% | 0% | 15% | 1% | 0% | 0% | 0% | 0% | **3%** |
| Genus | unclassified genus | 13% | 3% | 1% | 2% | 1% | 0% | 2% | 0% | 0% | 1% | 1% | 7% | 0% | 0% | 1% | **2%** |
| Genus | *Trichocoleus* | 0% | 2% | 1% | 1% | 0% | 1% | 3% | 0% | 4% | 1% | 4% | 0% | 0% | 0% | 9% | **2%** |
| Genus | unclassified genus | 0% | 3% | 0% | 0% | 0% | 1% | 1% | 0% | 2% | 1% | 1% | 4% | 1% | 12% | 1% | **2%** |
| Genus | *Scytonema* | 8% | 1% | 3% | 2% | 4% | 3% | 0% | 1% | 2% | 1% | 0% | 0% | 0% | 0% | 0% | **2%** |
| Genus | *Phormidesmis* | 0% | 0% | 0% | 0% | 0% | 16% | 0% | 0% | 0% | 0% | 0% | 7% | 0% | 0% | 1% | **2%** |
| Genus | unclassified genus | 0% | 0% | 0% | 0% | 0% | 0% | 2% | 2% | 12% | 0% | 0% | 4% | 0% | 0% | 1% | **1%** |
| Genus | unclassified genus | 18% | 0% | 0% | 0% | 0% | 0% | 0% | 0% | 0% | 0% | 0% | 0% | 0% | 0% | 0% | **1%** |
| Genus | unclassified genus | 0% | 11% | 0% | 0% | 0% | 0% | 0% | 0% | 0% | 0% | 0% | 4% | 0% | 0% | 1% | **1%** |
| Genus | <7% abund. | 43% | 13% | 10% | 15% | 17% | 16% | 1% | 1% | 4% | 1% | 4% | 11% | 1% | 12% | 2% | **10%** |

Supplement Table 6: Relative abundance within Phylum Archaea.

| Taxo-  nomic  rank | Taxo-  nomic  name | P-COLL | P-SMAL | P-TOBO | T-EAST | T-WEST | T-  TAYL | M-WELL | M-RABB | M-NORT | G-BASN | G-IBPE | G-SUMM | C-SAND | C-  CALI | C-GRAV | **Average** |
| --- | --- | --- | --- | --- | --- | --- | --- | --- | --- | --- | --- | --- | --- | --- | --- | --- | --- |
| Phylum | Euryarchae-ota | 96% | 100% | 100% | 100% | 100% | 100% | 100% | 100% | 100% | 100% | 100% | 98% | 99% | 100% | 99% | **99%** |
| Phylum | Nanoarchae-ota | 4% | 0% | 0% | 0% | 0% | 0% | 0% | 0% | 0% | 0% | 0% | 2% | 1% | 0% | 0% | **1%** |
| Phylum | Thaumarch-aeota | 0% | 0% | 0% | 0% | 0% | 0% | 0% | 0% | 0% | 0% | 0% | 0% | 0% | 0% | 0% | **0%** |
| Family | Nitroso-sphaeraceae | 96% | 100% | 100% | 100% | 100% | 100% | 100% | 100% | 100% | 100% | 100% | 98% | 99% | 100% | 99% | **99%** |
| Family | uncult. halo-archaeon | 2% | 0% | 0% | 0% | 0% | 0% | 0% | 0% | 0% | 0% | 0% | 1% | 0% | 0% | 0% | **0%** |
| Family | uncult. eury-archaeote | 1% | 0% | 0% | 0% | 0% | 0% | 0% | 0% | 0% | 0% | 0% | 0% | 0% | 0% | 0% | **0%** |
| Family | uncult. archaeon | 1% | 0% | 0% | 0% | 0% | 0% | 0% | 0% | 0% | 0% | 0% | 0% | 0% | 0% | 0% | **0%** |
| Family | Unknown | 0% | 0% | 0% | 0% | 0% | 0% | 0% | 0% | 0% | 0% | 0% | 1% | 0% | 0% | 0% | **0%** |
| Family | Methanobacteriaceae | 1% | 0% | 0% | 0% | 0% | 0% | 0% | 0% | 0% | 0% | 0% | 0% | 0% | 0% | 0% | **0%** |
| Genus | Cand. Nitro-sosphaera | 37% | 22% | 52% | 53% | 52% | 33% | 43% | 21% | 23% | 48% | 49% | 31% | 50% | 51% | 57% | **41%** |
| Genus | Unknown | 39% | 37% | 41% | 32% | 41% | 43% | 36% | 54% | 28% | 37% | 41% | 42% | 32% | 29% | 30% | **37%** |
| Genus | Cand. Nitro-cosmicus | 20% | 41% | 7% | 14% | 8% | 24% | 21% | 25% | 49% | 14% | 10% | 25% | 16% | 20% | 12% | **20%** |
| Genus | uncult. eury-archaeote | 1% | 0% | 0% | 0% | 0% | 0% | 0% | 0% | 0% | 0% | 0% | 1% | 0% | 0% | 1% | **0%** |
| Genus | uncult. halo-archaeon | 2% | 0% | 0% | 0% | 0% | 0% | 0% | 0% | 0% | 0% | 0% | 1% | 0% | 0% | 0% | **0%** |
| Genus | uncultured archaeon | 1% | 0% | 0% | 0% | 0% | 0% | 0% | 0% | 0% | 0% | 0% | 0% | 0% | 0% | 0% | **0%** |
| Genus | Methanobacterium | 1% | 0% | 0% | 0% | 0% | 0% | 0% | 0% | 0% | 0% | 0% | 0% | 0% | 0% | 0% | **0%** |

Supplement Table 7: Relative abundance within Phylum Fungi.

| Taxo-  nomic  rank | Taxo-  nomic  name | P-COLL | P-SMAL | P-TOBO | T-EAST | T-WEST | T-  TAYL | M-WELL | M-RABB | M-NORT | G-BASN | G-IBPE | G-SUMM | C-SAND | C-CALI | C-GRAV | **Average** |
| --- | --- | --- | --- | --- | --- | --- | --- | --- | --- | --- | --- | --- | --- | --- | --- | --- | --- |
| Phylum | Ascomycota | 76% | 55% | 62% | 68% | 80% | 68% | 84% | 76% | 85% | 74% | 64% | 63% | 77% | 79% | 22% | 69% |
| Phylum | Basidiomyco-ta | 15% | 41% | 6% | 20% | 11% | 20% | 10% | 15% | 9% | 12% | 21% | 15% | 14% | 9% | 71% | 19% |
| Phylum | Unknown phylum | 6% | 4% | 30% | 10% | 6% | 9% | 5% | 8% | 5% | 12% | 14% | 21% | 9% | 12% | 6% | 11% |
| Phylum | Mortierello-mycota | 2% | 1% | 0% | 1% | 2% | 0% | 0% | 0% | 0% | 1% | 0% | 0% | 0% | 0% | 0% | 0% |
| Phylum | Rozellomyco-ta | 0% | 0% | 1% | 1% | 1% | 0% | 0% | 0% | 0% | 1% | 0% | 0% | 0% | 0% | 0% | 0% |
| Phylum | Mucoromyco-ta | 0% | 0% | 0% | 0% | 0% | 1% | 0% | 0% | 0% | 0% | 0% | 0% | 0% | 0% | 0% | 0% |
| Phylum | <1% abund. | 0% | 0% | 0% | 0% | 0% | 0% | 0% | 0% | 0% | 0% | 0% | 0% | 0% | 0% | 0% | 0% |
| Class | Dothideo-mycetes | 34% | 36% | 20% | 45% | 38% | 18% | 47% | 31% | 63% | 39% | 27% | 33% | 51% | 48% | 11% | 36% |
| Class | Unknown  class | 10% | 6% | 38% | 18% | 12% | 17% | 26% | 31% | 19% | 32% | 34% | 37% | 23% | 21% | 11% | 22% |
| Class | Agarico-mycetes | 13% | 40% | 4% | 17% | 7% | 16% | 5% | 8% | 6% | 9% | 17% | 12% | 4% | 5% | 68% | 15% |
| Class | Eurotio-mycetes | 8% | 3% | 19% | 5% | 22% | 32% | 5% | 15% | 2% | 5% | 7% | 4% | 3% | 17% | 1% | 10% |
| Class | Sordario-mycetes | 16% | 11% | 13% | 7% | 6% | 2% | 11% | 8% | 6% | 7% | 6% | 7% | 8% | 4% | 4% | 8% |
| Class | Tremello-mycetes | 1% | 1% | 1% | 3% | 4% | 4% | 5% | 7% | 3% | 2% | 4% | 2% | 9% | 3% | 4% | 3% |
| Class | Pezizo-mycetes | 13% | 0% | 1% | 2% | 6% | 2% | 1% | 0% | 0% | 4% | 3% | 2% | 1% | 1% | 1% | 2% |
| Class | Leotio-mycetes | 1% | 1% | 1% | 2% | 1% | 0% | 0% | 0% | 0% | 0% | 0% | 1% | 0% | 2% | 0% | 1% |
| Class | Lecanoro-mycetes | 1% | 0% | 1% | 0% | 1% | 5% | 0% | 0% | 0% | 0% | 0% | 0% | 0% | 0% | 0% | 1% |
| Class | Mortierello-mycetes | 2% | 1% | 0% | 0% | 1% | 0% | 0% | 0% | 0% | 0% | 0% | 0% | 0% | 0% | 0% | 0% |
| Class | Orbilio-mycetes | 0% | 0% | 0% | 0% | 1% | 0% | 0% | 0% | 0% | 0% | 1% | 0% | 0% | 0% | 0% | 0% |
| Class | Mucoro-mycetes | 0% | 0% | 0% | 0% | 0% | 1% | 0% | 0% | 0% | 0% | 0% | 0% | 0% | 0% | 0% | 0% |
| Class | <1% abund. | 1% | 0% | 2% | 1% | 1% | 2% | 0% | 0% | 0% | 0% | 0% | 0% | 0% | 0% | 0% | 1% |
| Family | Unknown  family | 44% | 28% | 67% | 62% | 56% | 63% | 51% | 54% | 67% | 58% | 56% | 59% | 53% | 39% | 23% | 52% |
| Family | Didymella-ceae | 8% | 9% | 2% | 4% | 5% | 4% | 19% | 5% | 6% | 9% | 7% | 9% | 9% | 29% | 4% | 9% |
| Family | Sporormia-ceae | 3% | 5% | 5% | 4% | 8% | 1% | 3% | 5% | 7% | 12% | 5% | 3% | 14% | 7% | 2% | 6% |
| Family | Agaricaceae | 0% | 0% | 0% | 0% | 0% | 1% | 1% | 1% | 2% | 0% | 9% | 1% | 1% | 0% | 61% | 5% |
| Family | Chaetomia-ceae | 1% | 4% | 3% | 3% | 2% | 1% | 4% | 5% | 4% | 3% | 1% | 2% | 5% | 2% | 2% | 3% |
| Family | Filobasidia-ceae | 1% | 1% | 1% | 2% | 4% | 4% | 4% | 5% | 2% | 1% | 3% | 2% | 4% | 3% | 2% | 3% |
| Family | Nidulariaceae | 0% | 38% | 0% | 0% | 0% | 0% | 0% | 0% | 0% | 0% | 0% | 0% | 0% | 0% | 0% | 3% |
| Family | Pleospora-ceae | 6% | 4% | 2% | 2% | 2% | 1% | 4% | 1% | 2% | 2% | 3% | 3% | 4% | 1% | 1% | 3% |
| Family | Verrucaria-ceae | 0% | 0% | 4% | 0% | 5% | 20% | 0% | 0% | 0% | 0% | 0% | 0% | 0% | 0% | 0% | 2% |
| Family | Aspergilla-ceae | 1% | 3% | 6% | 1% | 2% | 0% | 2% | 3% | 1% | 2% | 0% | 2% | 1% | 0% | 0% | 2% |
| Family | Ceratobasidi-aceae | 3% | 0% | 0% | 1% | 2% | 0% | 0% | 2% | 0% | 1% | 4% | 3% | 1% | 0% | 4% | 1% |
| Family | Aureobasidi-aceae | 6% | 0% | 1% | 1% | 1% | 1% | 1% | 0% | 0% | 2% | 2% | 4% | 1% | 0% | 0% | 1% |
| Family | Coniothyria-ceae | 0% | 0% | 0% | 9% | 3% | 3% | 1% | 0% | 0% | 0% | 0% | 0% | 0% | 0% | 0% | 1% |
| Family | Trichocoma-ceae | 6% | 0% | 0% | 0% | 0% | 0% | 0% | 9% | 0% | 0% | 0% | 0% | 0% | 0% | 0% | 1% |
| Family | Onygena-ceae | 0% | 0% | 0% | 0% | 0% | 0% | 0% | 1% | 0% | 1% | 0% | 0% | 0% | 13% | 0% | 1% |
| Family | <1% abund. | 21% | 8% | 7% | 10% | 10% | 2% | 10% | 9% | 9% | 9% | 9% | 12% | 6% | 5% | 2% | 9% |
| Genus | Unknown | 65% | 36% | 71% | 70% | 67% | 68% | 61% | 62% | 74% | 67% | 67% | 76% | 63% | 55% | 30% | 62% |
| Genus | Phoma | 1% | 1% | 0% | 3% | 3% | 2% | 16% | 3% | 3% | 5% | 6% | 3% | 6% | 21% | 2% | 5% |
| Genus | Coprinus | 0% | 0% | 0% | 0% | 0% | 0% | 0% | 0% | 0% | 0% | 9% | 0% | 0% | 0% | 59% | 5% |
| Genus | Westerdy-kella | 2% | 5% | 2% | 1% | 6% | 0% | 1% | 3% | 5% | 10% | 3% | 2% | 10% | 0% | 1% | 3% |
| Genus | Cyathus | 0% | 38% | 0% | 0% | 0% | 0% | 0% | 0% | 0% | 0% | 0% | 0% | 0% | 0% | 0% | 3% |
| Genus | Naganishia | 1% | 1% | 1% | 2% | 3% | 3% | 4% | 4% | 2% | 1% | 3% | 0% | 4% | 2% | 2% | 2% |
| Genus | Placidium | 0% | 0% | 4% | 0% | 2% | 20% | 0% | 0% | 0% | 0% | 0% | 0% | 0% | 0% | 0% | 2% |
| Genus | Aspergillus | 1% | 3% | 6% | 0% | 2% | 0% | 2% | 3% | 1% | 2% | 0% | 2% | 1% | 0% | 0% | 2% |
| Genus | Coniothyrium | 1% | 0% | 4% | 1% | 1% | 1% | 0% | 0% | 0% | 0% | 1% | 1% | 2% | 6% | 0% | 1% |
| Genus | Subramaniula | 0% | 2% | 1% | 2% | 0% | 0% | 3% | 1% | 3% | 2% | 0% | 0% | 1% | 0% | 0% | 1% |
| Genus | Talaromyces | 0% | 0% | 0% | 9% | 3% | 3% | 1% | 0% | 0% | 0% | 0% | 0% | 0% | 0% | 0% | 1% |
| Genus | Preussia | 6% | 0% | 0% | 0% | 0% | 0% | 0% | 9% | 0% | 0% | 0% | 0% | 0% | 0% | 0% | 1% |
| Genus | Macroventu-ria | 65% | 36% | 71% | 70% | 67% | 68% | 61% | 62% | 74% | 67% | 67% | 76% | 63% | 55% | 30% | 62% |
| Genus | Aureobasi-dium | 1% | 1% | 0% | 3% | 3% | 2% | 16% | 3% | 3% | 5% | 6% | 3% | 6% | 21% | 2% | 5% |
| Genus | Ascochyta | 0% | 0% | 0% | 0% | 0% | 0% | 0% | 0% | 0% | 0% | 9% | 0% | 0% | 0% | 59% | 5% |
| Genus | Paraphaeo-sphaeria | 2% | 5% | 2% | 1% | 6% | 0% | 1% | 3% | 5% | 10% | 3% | 2% | 10% | 0% | 1% | 3% |
| Genus | Articulospora | 0% | 38% | 0% | 0% | 0% | 0% | 0% | 0% | 0% | 0% | 0% | 0% | 0% | 0% | 0% | 3% |
| Genus | Chaetomium | 1% | 1% | 1% | 2% | 3% | 3% | 4% | 4% | 2% | 1% | 3% | 0% | 4% | 2% | 2% | 2% |
| Genus | Albifimbria | 0% | 0% | 4% | 0% | 2% | 20% | 0% | 0% | 0% | 0% | 0% | 0% | 0% | 0% | 0% | 2% |
| Genus | Geastrum | 1% | 3% | 6% | 0% | 2% | 0% | 2% | 3% | 1% | 2% | 0% | 2% | 1% | 0% | 0% | 2% |
| Genus | Ceratobasi-dium | 1% | 0% | 4% | 1% | 1% | 1% | 0% | 0% | 0% | 0% | 1% | 1% | 2% | 6% | 0% | 1% |
| Genus | Paraconio-thyrium | 0% | 2% | 1% | 2% | 0% | 0% | 3% | 1% | 3% | 2% | 0% | 0% | 1% | 0% | 0% | 1% |
| Genus | Entoloma | 0% | 0% | 0% | 9% | 3% | 3% | 1% | 0% | 0% | 0% | 0% | 0% | 0% | 0% | 0% | 1% |
| Genus | Acremonium | 6% | 0% | 0% | 0% | 0% | 0% | 0% | 9% | 0% | 0% | 0% | 0% | 0% | 0% | 0% | 1% |
| Genus | <1% abund. | 19% | 16% | 21% | 22% | 17% | 16% | 17% | 21% | 16% | 20% | 14% | 22% | 16% | 12% | 10% | 17% |

Supplement Table 8: Linear model results testing Vegetation Zone, Ecological Site, Landform, and Landscape, on relative abundance of Bacteria phyla. Tukey method was used for mean comparison.

| **Taxonomic rank** | **Taxonomic name** | **Overall Abundance Rank** | **D.F.** | **Global p-value** | **Factor** | **Factor levels; means with the same letter are not different**  **at ɑ = 0.05** |
| --- | --- | --- | --- | --- | --- | --- |
| Phylum | Proteobacteria | 1 | 9 | 0.018 | Vegetation zone | Tarbush (A), Creosote (AB), Grass (AB), Mesquite (AB), Playa (B) |
| Phylum | Proteobacteria | 1 | 9 | 0.024 | Ecological Site | Loamy (A), Gravelly (AB), Sandy (AB), Gravelly sand (AB), Playa (B) |
| Phylum | Proteobacteria | 1 | 10 | 0.012 | Landform | Alluvial Flat (A), Fan Piedmont (AB), Alluvial Plain (AB), Playa (B) |
| Phylum | Proteobacteria | 1 | 12 | 0.663 | Landscape | Piedmont Slope (A), Basin Floor (A) |
| Phylum | Actinobacteria | 2 | 9 | 0.050 | Vegetation zone | Playa (A), Grass (A), Mesquite (A), Tarbush (A), Creosote (A) |
| Phylum | Actinobacteria | 2 | 9 | 0.542 | Ecological Site | Playa (A), Sandy (A), Gravelly sand (A), Loamy (A), Gravelly (A) |
| Phylum | Actinobacteria | 2 | 10 | 0.535 | Landform | Playa (A), Alluvial Plain (A), Fan Piedmont (A), Alluvial Flat (A) |
| Phylum | Actinobacteria | 2 | 12 | 0.866 | Landscape | Basin Floor (A), Piedmont Slope (A) |
| Phylum | Chloroflexi | 3 | 9 | 0.008 | Ecological Site | Gravelly sand (A), Sandy (A), Playa (AB), Gravelly (AB), Loamy (B) |
| Phylum | Chloroflexi | 3 | 9 | 0.046 | Vegetation zone | Mesquite (A), Grass (AB), Playa (AB), Creosote (AB), Tarbush (B) |
| Phylum | Chloroflexi | 3 | 10 | 0.023 | Landform | Alluvial Plain (A), Playa (AB), Fan Piedmont (AB), Alluvial Flat (B) |
| Phylum | Chloroflexi | 3 | 12 | 0.539 | Landscape | Basin Floor (A), Piedmont Slope (A) |
| Phylum | Acidobacteria | 4 | 9 | 0.135 | Vegetation zone | Tarbush (A), Creosote (A), Mesquite (A), Playa (A), Grass (A) |
| Phylum | Acidobacteria | 4 | 9 | 0.709 | Ecological Site | Loamy (A), Gravelly (A), Sandy (A), Gravelly sand (A), Playa (A) |
| Phylum | Acidobacteria | 4 | 10 | 0.333 | Landform | Alluvial Flat (A), Fan Piedmont (A), Alluvial Plain (A), Playa (A) |
| Phylum | Acidobacteria | 4 | 12 | 0.842 | Landscape | Basin Floor (A), Piedmont Slope (A) |
| Phylum | Bacteroidetes | 5 | 9 | 0.088 | Vegetation zone | Tarbush (A), Creosote (A), Playa (A), Mesquite (A), Grass (A) |
| Phylum | Bacteroidetes | 5 | 9 | 0.292 | Ecological Site | Loamy (A), Gravelly (A), Playa (A), Gravelly sand (A), Sandy (A) |
| Phylum | Bacteroidetes | 5 | 10 | 0.190 | Landform | Alluvial Flat (A), Fan Piedmont (A), Playa (A), Alluvial Plain (A) |
| Phylum | Bacteroidetes | 5 | 12 | 0.539 | Landscape | Piedmont Slope (A), Basin Floor (A) |
| Phylum | Gemmatimonadetes | 6 | 9 | 0.409 | Vegetation zone | Playa (A), Creosote (A), Tarbush (A), Mesquite (A), Grass (A) |
| Phylum | Gemmatimonadetes | 6 | 9 | 0.632 | Ecological Site | Playa (A), Gravelly sand (A), Gravelly (A), Loamy (A), Sandy (A) |
| Phylum | Gemmatimonadetes | 6 | 10 | 0.521 | Landform | Playa (A), Alluvial Flat (A), Fan Piedmont (A), Alluvial Plain (A) |
| Phylum | Gemmatimonadetes | 6 | 12 | 0.835 | Landscape | Basin Floor (A), Piedmont Slope (A) |
| Phylum | Planctomycetes | 7 | 9 | 0.011 | Ecological Site | Sandy (A), Gravelly sand (A), Gravelly (AB), Playa (AB), Loamy (B) |
| Phylum | Planctomycetes | 7 | 9 | 0.030 | Vegetation zone | Mesquite (A), Creosote (AB), Playa (AB), Grass (AB), Tarbush (B) |
| Phylum | Planctomycetes | 7 | 10 | 0.008 | Landform | Alluvial Plain (A), Playa (AB), Fan Piedmont (A), Alluvial Flat (B) |
| Phylum | Planctomycetes | 7 | 12 | 0.438 | Landscape | Basin Floor (A), Piedmont Slope (A) |
| Phylum | Cyanobacteria | 8 | 9 | 0.011 | Vegetation zone | Playa (A), Creosote (A), Grass (A), Mesquite (A), Tarbush (B) |
| Phylum | Cyanobacteria | 8 | 9 | 0.105 | Ecological Site | Gravelly (A), Playa (A), Gravelly sand (A), Sandy (A), Loamy (A) |
| Phylum | Cyanobacteria | 8 | 10 | 0.002 | Landform | Playa (A), Fan Piedmont (A), Alluvial Plain (A), Alluvial Flat (B) |
| Phylum | Cyanobacteria | 8 | 12 | 0.468 | Landscape | Piedmont Slope (A), Basin Floor (A) |
| Phylum | Verrucomicrobia | 10 | 9 | 0.526 | Vegetation zone | Mesquite (A), Tarbush (A), Creosote (A), Grass (A), Playa (A) |
| Phylum | Verrucomicrobia | 10 | 9 | 0.807 | Ecological Site | Sandy (A), Loamy (A), Gravelly sand (A), Gravelly (A), Playa (A) |
| Phylum | Verrucomicrobia | 10 | 10 | 0.521 | Landform | Alluvial Flat (A), Alluvial Plain (A), Fan Piedmont (A), Playa (A) |
| Phylum | Verrucomicrobia | 10 | 12 | 0.287 | Landscape | Basin Floor (A), Piedmont Slope (A) |
| Phylum | Armatimonadetes | 11 | 9 | 0.007 | Ecological Site | Playa (A), Sandy (B), Gravelly sand (AB), Loamy (B), Gravelly (B) |
| Phylum | Armatimonadetes | 11 | 9 | 0.033 | Vegetation zone | Playa (A), Mesquite (AB), Creosote (B), Tarbush (B), Grass (B) |
| Phylum | Armatimonadetes | 11 | 10 | 0.004 | Landform | Playa (A), Alluvial Plain (B), Alluvial Flat (B), Fan Piedmont (B) |
| Phylum | Armatimonadetes | 11 | 12 | 0.026 | Landscape | Basin Floor (A), Piedmont Slope (B) |

Supplement Table 9: Linear model results testing Vegetation Zone, Ecological Site, Landform, and Landscape, on relative abundance of Cyanobacteria families. Tukey method was used for mean comparison.

| **Taxonomic rank** | **Taxonomic name** | **Overall Abundance Rank** | **D.F.** | **Global p-value** | **Factor** | **Factor Levels; means with the same letter are not different at ɑ = 0.05** |
| --- | --- | --- | --- | --- | --- | --- |
| Family | Microcoleaceae | 1 | 9 | 0.018 | Vegetation zone | Playa (A), Tarbush (AB), Grass (AB), Mesquite (B), Creosote (B) |
| Family | Microcoleaceae | 1 | 9 | 0.180 | Ecological Site | Playa (A), Loamy (A), Sandy (A), Gravelly (A), Gravelly sand (A) |
| Family | Microcoleaceae | 1 | 10 | 0.076 | Landform | Playa (A), Alluvial Flat (A), Alluvial Plain (A), Fan Piedmont (A) |
| Family | Microcoleaceae | 1 | 12 | 0.336 | Landscape | Basin Floor (A), Piedmont Slope (A) |
| Family | Coleofasciculaceae | 2 | 9 | 0.001 | Vegetation zone | Creosote (A), Mesquite (A), Playa (AB), Grass (B), Tarbush (B) |
| Family | Coleofasciculaceae | 2 | 9 | 0.105 | Ecological Site | Gravelly (A), Gravelly sand (A), Playa (A), Sandy (A), Loamy (A) |
| Family | Coleofasciculaceae | 2 | 10 | 0.177 | Landform | Fan Piedmont (A), Playa (A), Alluvial Plain (A), Alluvial Flat (A) |
| Family | Coleofasciculaceae | 2 | 12 | 0.578 | Landscape | Piedmont Slope (A), Basin Floor (A) |
| Family | Leptolyngbyaceae | 3 | 9 | 0.218 | Vegetation zone | Mesquite (A), Creosote (A), Grass (A), Tarbush (A), Playa (A) |
| Family | Leptolyngbyaceae | 3 | 9 | 0.315 | Ecological Site | Sandy (A), Gravelly (A), Gravelly sand (A), Loamy (A), Playa (A) |
| Family | Leptolyngbyaceae | 3 | 10 | 0.126 | Landform | Alluvial Plain (A), Fan Piedmont (A), Alluvial Flat (A), Playa (A) |
| Family | Leptolyngbyaceae | 3 | 12 | 0.761 | Landscape | Basin Floor (A), Piedmont Slope (A) |
| Family | unclassified Cyanobacteria family | 4 | 9 | 0.605 | Ecological Site | Gravelly sand (A), Loamy (A), Sandy (A), Playa (A), Gravelly (A) |
| Family | unclassified Cyanobacteria family | 4 | 9 | 0.976 | Vegetation zone | Grass (A), Tarbush (A), Creosote (A), Mesquite (A), Playa (A) |
| Family | unclassified Cyanobacteria family | 4 | 10 | 0.862 | Landform | Fan Piedmont (A), Alluvial Flat (A), Alluvial Plain (A), Playa (A) |
| Family | unclassified Cyanobacteria family | 4 | 12 | 0.313 | Landscape | Piedmont Slope (A), Basin Floor (A) |
| Family | Gomontiellaceae | 5 | 9 | 0.450 | Vegetation zone | Playa (A), Tarbush (A), Grass (A), Creosote (A), Mesquite (A) |
| Family | Gomontiellaceae | 5 | 9 | 0.513 | Ecological Site | Playa (A), Loamy (A), Gravelly sand (A), Sandy (A), Gravelly (A) |
| Family | Gomontiellaceae | 5 | 10 | 0.514 | Landform | Playa (A), Alluvial Flat (A), Fan Piedmont (A), Alluvial Plain (A) |
| Family | Gomontiellaceae | 5 | 12 | 0.998 | Landscape | Piedmont Slope (A), Basin Floor (A) |
| Family | Oscillatoriaceae | 6 | 9 | 0.128 | Vegetation zone | Creosote (A), Mesquite (A), Tarbush (A), Grass (A), Playa (A) |
| Family | Oscillatoriaceae | 6 | 9 | 0.135 | Ecological Site | Gravelly (A), Sandy (A), Loamy (A), Gravelly sand (A), Playa (A) |
| Family | Oscillatoriaceae | 6 | 10 | 0.081 | Landform | Alluvial Plain (A), Alluvial Flat (A), Fan Piedmont (A), Playa (A) |
| Family | Oscillatoriaceae | 6 | 12 | 0.536 | Landscape | Piedmont Slope (A), Basin Floor (A) |
| Family | Trichocoleusaceae | 8 | 9 | 0.374 | Ecological Site | Gravelly sand (A), Loamy (A), Playa (A), Sandy (A), Gravelly (A) |
| Family | Trichocoleusaceae | 8 | 9 | 0.820 | Vegetation zone | Tarbush (A), Playa (A), Grass (A), Mesquite (A), Creosote (A) |
| Family | Trichocoleusaceae | 8 | 10 | 0.688 | Landform | Alluvial Flat (A), Playa (A), Fan Piedmont (A), Alluvial Plain (A) |
| Family | Trichocoleusaceae | 8 | 12 | 0.952 | Landscape | Basin Floor (A), Piedmont Slope (A) |
| Family | Chroococcidiopsidaceae | 9 | 9 | 0.314 | Ecological Site | Loamy (A), Sandy (A), Playa (A), Gravelly sand (A), Gravelly (A) |
| Family | Chroococcidiopsidaceae | 9 | 9 | 0.651 | Vegetation zone | Tarbush (A), Mesquite (A), Playa (A), Grass (A), Creosote (A) |
| Family | Chroococcidiopsidaceae | 9 | 10 | 0.502 | Landform | Alluvial Flat (A), Alluvial Plain (A), Playa (A), Fan Piedmont (A) |
| Family | Chroococcidiopsidaceae | 9 | 12 | 0.201 | Landscape | Basin Floor (A), Piedmont Slope (A) |
| Family | Scytonemataceae | 10 | 9 | 0.100 | Vegetation zone | Creosote (A), Grass (A), Mesquite (A), Tarbush (A), Playa (A) |
| Family | Scytonemataceae | 10 | 9 | 0.172 | Ecological Site | Gravelly (A), Gravelly sand (A), Sandy (A), Loamy (A), Playa (A) |
| Family | Scytonemataceae | 10 | 10 | 0.045 | Landform | Fan Piedmont (A), Alluvial Plain (A), Alluvial Flat (A), Playa (A) |
| Family | Scytonemataceae | 10 | 12 | 0.171 | Landscape | Piedmont Slope (A), Basin Floor (A) |
| Family | Nostocaceae | 11 | 9 | 0.191 | Vegetation zone | Creosote (A), Grass (A), Mesquite (A), Tarbush (A), Playa (A) |
| Family | Nostocaceae | 11 | 9 | 0.191 | Ecological Site | Gravelly sand (A), Gravelly (A), Sandy (A), Loamy (A), Playa (A) |
| Family | Nostocaceae | 11 | 10 | 0.092 | Landform | Alluvial Plain (A), Fan Piedmont (A), Alluvial Flat (A), Playa (A) |
| Family | Nostocaceae | 11 | 12 | 0.401 | Landscape | Piedmont Slope (A), Basin Floor (A) |
| Family | unclassified Oscillatoriales family | 13 | 9 | 0.222 | Ecological Site | Sandy (A), Loamy (A), Gravelly (A), Gravelly sand (A), Playa (A) |
| Family | unclassified Oscillatoriales family | 13 | 9 | 0.270 | Vegetation zone | Mesquite (A), Tarbush (A), Creosote (A), Grass (A), Playa (A) |
| Family | unclassified Oscillatoriales family | 13 | 10 | 0.145 | Landform | Alluvial Plain (A), Alluvial Flat (A), Fan Piedmont (A), Playa (A) |
| Family | unclassified Oscillatoriales family | 13 | 12 | 0.711 | Landscape | Piedmont Slope (A), Basin Floor (A) |

Supplement Table 10: Linear model results testing Vegetation Zone, Ecological Site, Landform, and Landscape, on relative abundance of Archaea genera. Tukey method was used for mean comparison.

| **Taxonomic rank** | **Taxonomic name** | **Overall Abundance Rank** | **D.F.** | **Global p-value** | **Factor** | **Factor Levels; means with the same letter are not different at ɑ = 0.05** |
| --- | --- | --- | --- | --- | --- | --- |
| Genus | Candidatus Nitrososphaera | 1 | 9 | 0.073 | Vegetation zone | Mesquite (A), Playa (A), Grass (A), Tarbush (A), Creosote (A) |
| Genus | Candidatus Nitrososphaera | 1 | 9 | 0.206 | Ecological Site | Playa (A), Sandy (A), Gravelly sand (A), Loamy (A), Gravelly (A) |
| Genus | Candidatus Nitrososphaera | 1 | 10 | 0.197 | Landform | Playa (A), Alluvial Plain (A), Alluvial Flat (A), Fan Piedmont (A) |
| Genus | Candidatus Nitrososphaera | 1 | 12 | 0.277 | Landscape | Basin Floor (A), Piedmont Slope (A) |
| Genus | Candidatus Nitrocosmicus | 3 | 9 | 0.225 | Vegetation zone | Tarbush (A), Creosote (A), Grass (A), Playa (A), Mesquite (A) |
| Genus | Candidatus Nitrocosmicus | 3 | 9 | 0.519 | Ecological Site | Loamy (A), Gravelly (A), Gravelly sand (A), Sandy (A), Playa (A) |
| Genus | Candidatus Nitrocosmicus | 3 | 10 | 0.377 | Landform | Alluvial Flat (A), Fan Piedmont (A), Alluvial Plain (A), Playa (A) |
| Genus | Candidatus Nitrocosmicus | 3 | 12 | 0.463 | Landscape | Piedmont Slope (A), Basin Floor (A) |
| Genus | uncultured euryarchaeote | 4 | 9 | 0.132 | Vegetation zone | Tarbush (A), Mesquite (A), Creosote (A), Playa (A), Grass (A) |
| Genus | uncultured euryarchaeote | 4 | 9 | 0.140 | Ecological Site | Loamy (A), Sandy (A), Gravelly (A), Playa (A), Gravelly sand (A) |
| Genus | uncultured euryarchaeote | 4 | 10 | 0.052 | Landform | Alluvial Flat (A), Alluvial Plain (AB), Playa (AB), Fan Piedmont (B) |
| Genus | uncultured euryarchaeote | 4 | 12 | 0.206 | Landscape | Basin Floor (A), Piedmont Slope (A) |
| Genus | uncultured haloarchaeon | 5 | 9 | 0.233 | Ecological Site | Loamy (A), Gravelly (A), Sandy (A), Gravelly sand (A), Playa (A) |
| Genus | uncultured haloarchaeon | 5 | 9 | 0.369 | Vegetation zone | Tarbush (A), Creosote (A), Mesquite (A), Grass (A), Playa (A) |
| Genus | uncultured haloarchaeon | 5 | 10 | 0.277 | Landform | Alluvial Flat (A), Alluvial Plain (A), Fan Piedmont (A), Playa (A) |
| Genus | uncultured haloarchaeon | 5 | 12 | 0.749 | Landscape | Piedmont Slope (A), Basin Floor (A) |
| Genus | uncultured archaeon | 6 | 9 | 0.145 | Vegetation zone | Tarbush (A), Mesquite (A), Grass (A), Creosote (A), Playa (A) |
| Genus | uncultured archaeon | 6 | 9 | 0.170 | Ecological Site | Loamy (A), Sandy (A), Gravelly sand (A), Gravelly (A), Playa (A) |
| Genus | uncultured archaeon | 6 | 10 | 0.077 | Landform | Alluvial Flat (A), Alluvial Plain (A), Fan Piedmont (A), Playa (A) |
| Genus | uncultured archaeon | 6 | 12 | 0.686 | Landscape | Piedmont Slope (A), Basin Floor (A) |
| Genus | Methanobacterium | 7 | 9 | 0.190 | Vegetation zone | Creosote (A), Mesquite (A), Tarbush (A), Grass (A), Playa (A) |
| Genus | Methanobacterium | 7 | 9 | 0.190 | Ecological Site | Gravelly (A), Sandy (A), Loamy (A), Gravelly sand (A), Playa (A) |
| Genus | Methanobacterium | 7 | 10 | 0.091 | Landform | Alluvial Flat (A), Alluvial Plain (A), Fan Piedmont (A), Playa (A) |
| Genus | Methanobacterium | 7 | 12 | 0.408 | Landscape | Piedmont Slope (A), Basin Floor (A) |

Supplement Table 11: Linear model results testing Vegetation Zone, Ecological Site, Landform, and Landscape, on relative abundance of Fungi classes. Tukey method was used for mean comparison.

| **Taxonomic rank** | **Taxonomic name** | **Overall Abundance Rank** | **D.F.** | **Global p-value** | **Factor** | **Factor Levels; means with the same letter are not different at ɑ = 0.05** |
| --- | --- | --- | --- | --- | --- | --- |
| Class | Dothideomycetes | 1 | 9 | 0.787 | Vegetation zone | Grass (A), Tarbush (A), Playa (A), Creosote (A), Mesquite (A) |
| Class | Dothideomycetes | 1 | 9 | 0.862 | Ecological Site | Gravelly (A), Loamy (A), Playa (A), Sandy (A), Gravelly sand (A) |
| Class | Dothideomycetes | 1 | 10 | 0.887 | Landform | Alluvial Flat (A), Playa (A), Fan Piedmont (A), Alluvial Plain (A) |
| Class | Dothideomycetes | 1 | 12 | 0.376 | Landscape | Piedmont Slope (A), Basin Floor (A) |
| Class | Agaricomycetes | 3 | 9 | 0.353 | Ecological Site | Gravelly sand (A), Sandy (A), Loamy (A), Playa (A), Gravelly (A) |
| Class | Agaricomycetes | 3 | 9 | 0.677 | Vegetation zone | Mesquite (A), Grass (A), Tarbush (A), Creosote (A), Playa (A) |
| Class | Agaricomycetes | 3 | 10 | 0.704 | Landform | Alluvial Plain (A), Alluvial Flat (A), Fan Piedmont (A), Playa (A) |
| Class | Agaricomycetes | 3 | 12 | 0.620 | Landscape | Basin Floor (A), Piedmont Slope (A) |
| Class | Eurotiomycetes | 4 | 9 | 0.293 | Vegetation zone | Grass (A), Playa (A), Creosote (A), Mesquite (A), Tarbush (A) |
| Class | Eurotiomycetes | 4 | 9 | 0.540 | Ecological Site | Gravelly sand (A), Playa (A), Sandy (A), Gravelly (A), Loamy (A) |
| Class | Eurotiomycetes | 4 | 10 | 0.159 | Landform | Playa (A), Fan Piedmont (A), Alluvial Plain (A), Alluvial Flat (A) |
| Class | Eurotiomycetes | 4 | 12 | 0.684 | Landscape | Basin Floor (A), Piedmont Slope (A) |
| Class | Sordariomycetes | 5 | 9 | 0.009 | Ecological Site | Gravelly (A), Loamy (A), Gravelly sand (AB), Sandy (AB), Playa (B) |
| Class | Sordariomycetes | 5 | 9 | 0.013 | Vegetation zone | Creosote (A), Tarbush (A), Grass (A), Mesquite (AB), Playa (B) |
| Class | Sordariomycetes | 5 | 10 | 0.009 | Landform | Alluvial Flat (A), Fan Piedmont (A), Alluvial Plain (AB), Playa (B) |
| Class | Sordariomycetes | 5 | 12 | 0.043 | Landscape | Piedmont Slope (A), Basin Floor (B) |
| Class | Tremellomycetes | 6 | 9 | 0.176 | Vegetation zone | Playa (A), Grass (A), Tarbush (A), Mesquite (A), Creosote (A) |
| Class | Tremellomycetes | 6 | 9 | 0.217 | Ecological Site | Playa (A), Loamy (A), Gravelly (A), Sandy (A), Gravelly sand (A) |
| Class | Tremellomycetes | 6 | 10 | 0.330 | Landform | Playa (A), Alluvial Flat (A), Fan Piedmont (A), Alluvial Plain (A) |
| Class | Tremellomycetes | 6 | 12 | 0.623 | Landscape | Basin Floor (A), Piedmont Slope (A) |
| Class | Pezizomycetes | 7 | 9 | 0.292 | Vegetation zone | Mesquite (A), Creosote (A), Grass (A), Tarbush (A), Playa (A) |
| Class | Pezizomycetes | 7 | 9 | 0.345 | Ecological Site | Gravelly (A), Sandy (A), Gravelly sand (A), Loamy (A), Playa (A) |
| Class | Pezizomycetes | 7 | 10 | 0.258 | Landform | Alluvial Plain (A), Fan Piedmont (A), Alluvial Flat (A), Playa (A) |
| Class | Pezizomycetes | 7 | 12 | 0.498 | Landscape | Piedmont Slope (A), Basin Floor (A) |
| Class | Leotiomycetes | 8 | 9 | 0.268 | Vegetation zone | Mesquite (A), Grass (A), Creosote (A), Playa (A), Tarbush (A) |
| Class | Leotiomycetes | 8 | 9 | 0.427 | Ecological Site | Sandy (A), Gravelly sand (A), Gravelly (A), Loamy (A), Playa (A) |
| Class | Leotiomycetes | 8 | 10 | 0.162 | Landform | Alluvial Plain (A), Fan Piedmont (A), Playa (A), Alluvial Flat (A) |
| Class | Leotiomycetes | 8 | 12 | 0.691 | Landscape | Piedmont Slope (A), Basin Floor (A) |
| Class | Lecanoromycetes | 9 | 9 | 0.274 | Vegetation zone | Creosote (A), Mesquite (A), Grass (A), Playa (A), Tarbush (A) |
| Class | Lecanoromycetes | 9 | 9 | 0.442 | Ecological Site | Gravelly (A), Sandy (A), Gravelly sand (A), Playa (A), Loamy (A) |
| Class | Lecanoromycetes | 9 | 10 | 0.146 | Landform | Alluvial Plain (A), Fan Piedmont (A), Playa (A), Alluvial Flat (A) |
| Class | Lecanoromycetes | 9 | 12 | 0.338 | Landscape | Basin Floor (A), Piedmont Slope (A) |
| Class | Mortierellomycetes | 11 | 9 | 0.073 | Vegetation zone | Mesquite (A), Creosote (A), Grass (A), Tarbush (A), Playa (A) |
| Class | Mortierellomycetes | 11 | 9 | 0.131 | Ecological Site | Gravelly (A), Sandy (A), Gravelly sand (A), Loamy (A), Playa (A) |
| Class | Mortierellomycetes | 11 | 10 | 0.044 | Landform | Alluvial Plain (AB), Fan Piedmont (A), Alluvial Flat (AB), Playa (B) |
| Class | Mortierellomycetes | 11 | 12 | 0.118 | Landscape | Piedmont Slope (A), Basin Floor (A) |
| Class | Orbiliomycetes | 12 | 9 | 0.339 | Vegetation zone | Creosote (A), Mesquite (A), Playa (A), Grass (A), Tarbush (A) |
| Class | Orbiliomycetes | 12 | 9 | 0.783 | Ecological Site | Gravelly (A), Gravelly sand (A), Playa (A), Sandy (A), Loamy (A) |
| Class | Orbiliomycetes | 12 | 10 | 0.346 | Landform | Fan Piedmont (A), Playa (A), Alluvial Plain (A), Alluvial Flat (A) |
| Class | Orbiliomycetes | 12 | 12 | 0.204 | Landscape | Piedmont Slope (A), Basin Floor (A) |
| Class | Mucoromycetes | 13 | 9 | 0.492 | Vegetation zone | Creosote (A), Mesquite (A), Grass (A), Playa (A), Tarbush (A) |
| Class | Mucoromycetes | 13 | 9 | 0.690 | Ecological Site | Gravelly (A), Sandy (A), Playa (A), Gravelly sand (A), Loamy (A) |
| Class | Mucoromycetes | 13 | 10 | 0.307 | Landform | Alluvial Plain (A), Fan Piedmont (A), Playa (A), Alluvial Flat (A) |
| Class | Mucoromycetes | 13 | 12 | 0.274 | Landscape | Basin Floor (A), Piedmont Slope (A) |

| Variable | Total Bacteria | | Cyanobacteria | | Archaea | | Fungi | | Notes |
| --- | --- | --- | --- | --- | --- | --- | --- | --- | --- |
|  | R^2^ | p_value | R^2^ | p_value | R^2^ | p_value | R^2^ | p_value |  |
| **% Clay** | 0.885 | **0.001** | 0.716 | **0.001** | 0.748 | **0.001** | 0.488 | **0.001** | Significant for all groups |
| **EC** | 0.550 | **0.001** | 0.445 | **0.001** | 0.428 | **0.002** | 0.582 | **0.001** | Significant for all groups |
| Gravel | 0.154 | 0.091 | 0.348 | **0.004** | 0.383 | **0.001** | 0.151 | 0.130 | not significant for Fungi; only marginal for Total bacteria |
| pH | 0.523 | **0.001** | 0.400 | **0.001** | 0.408 | **0.001** | 0.188 | 0.089 | not significant for Fungi |
| **% Sand** | 0.929 | **0.001** | 0.730 | **0.001** | 0.765 | **0.001** | 0.595 | **0.001** | Significant for all groups |
| Grass biomass | 0.029 | 0.713 | 0.124 | 0.186 | 0.019 | 0.792 | 0.000 | 1.000 | Not significant for any group |
| **Shrub biomass** | 0.328 | **0.004** | 0.291 | **0.008** | 0.327 | **0.003** | 0.696 | **0.001** | Significant for all groups |
| Lichen crust cover | 0.195 | 0.080 | 0.146 | 0.150 | 0.167 | 0.095 | 0.392 | **0.001** | Significant for Fungi; marginal for Total Bacteria and Archaea |
| Maximum Daily Air Temperature | 0.176 | 0.107 | 0.047 | 0.530 | 0.116 | 0.200 | 0.047 | 0.547 | Not significant for any group |
| **Soil Moisture** | 0.534 | **0.001** | 0.452 | **0.002** | 0.504 | **0.001** | 0.608 | **0.001** | Significant for all groups |

Supplement Table 12: Results of the environmental fit analysis (R^2^ and p-values).

Supplemental Figure 1: Composition of selected taxa. Relative abundance data also appear in Supplemental Table 3-6.


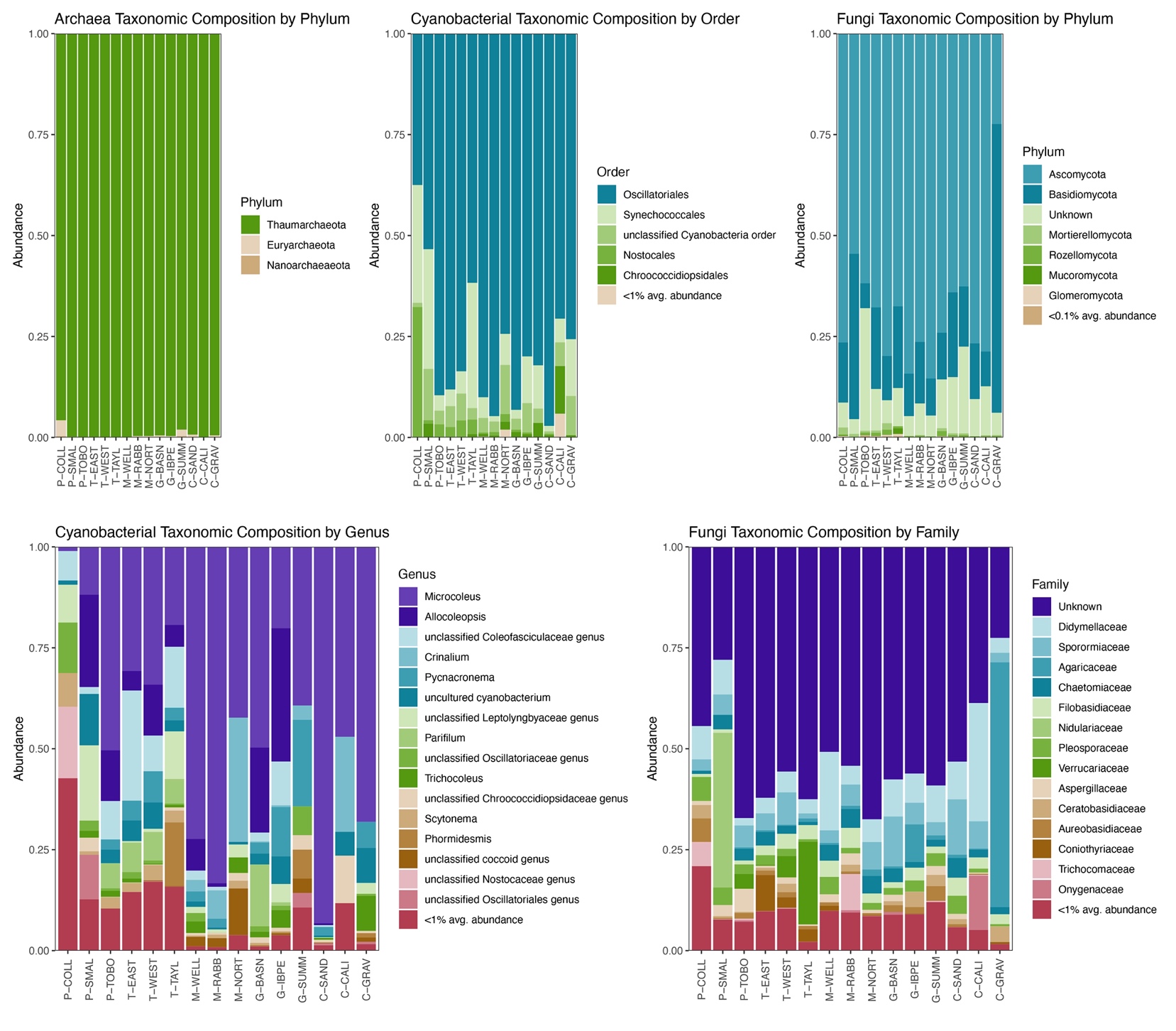


Supplemental Figure 2: Polynomial regression results relating microbial observed richness to continuous environmental variables. Undisturbed and disturbed samples were plotted, and color coded by Landform. Linear, quadratic and cubic trends were examined. Trendlines are shown only for significant relationships at the highest polynomial order. Panels with no trendline had no significant linear, quadratic, or cubic relationships.


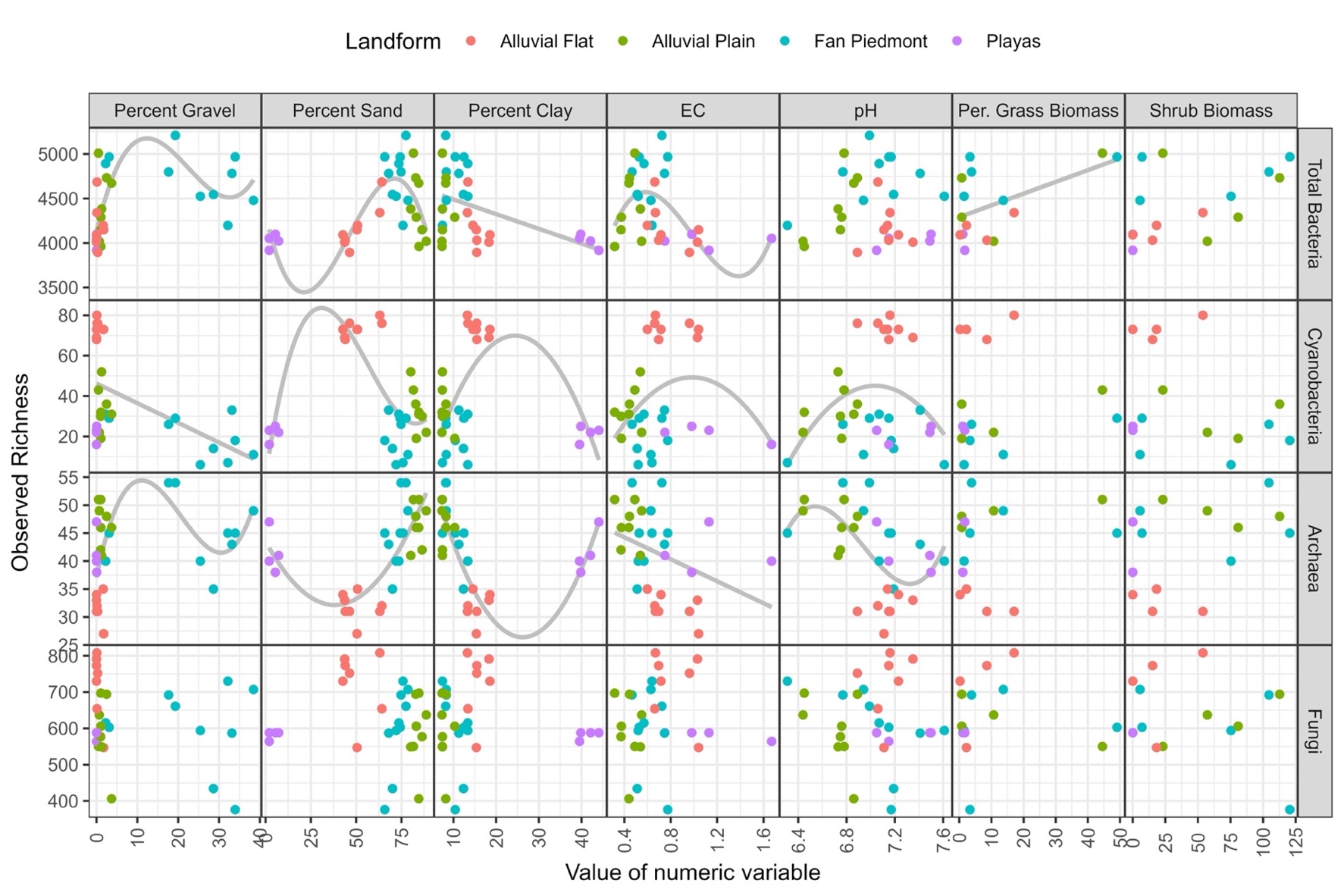

Supplement: Supplementary file 1 [file Data_Sheet_1.docx]
